# The Spectrin cytoskeleton regulates the Hippo signalling pathway

**DOI:** 10.15252/embj.201489642

**Published:** 2015-02-23

**Authors:** Georgina C Fletcher, Ahmed Elbediwy, Ichha Khanal, Paulo S Ribeiro, Nic Tapon, Barry J Thompson

**Affiliations:** 1 Epithelial Biology Laboratory, Cancer Research UK – London Research InstituteLondon, UK; 2 Apoptosis and Cell Proliferation Laboratory, Cancer Research UK – London Research InstituteLondon, UK; 3 Barts Cancer Institute, Queen Mary University of LondonLondon, UK

**Keywords:** cytoskeleton, Hippo signalling, Spectrin, YAP

## Abstract

The Spectrin cytoskeleton is known to be polarised in epithelial cells, yet its role remains poorly understood. Here, we show that the Spectrin cytoskeleton controls Hippo signalling. In the developing *Drosophila* wing and eye, loss of apical Spectrins (alpha/beta-heavy dimers) produces tissue overgrowth and mis-regulation of Hippo target genes, similar to loss of Crumbs (Crb) or the FERM-domain protein Expanded (Ex). Apical beta-heavy Spectrin binds to Ex and co-localises with it at the apical membrane to antagonise Yki activity. Interestingly, in both the ovarian follicular epithelium and intestinal epithelium of *Drosophila*, apical Spectrins and Crb are dispensable for repression of Yki, while basolateral Spectrins (alpha/beta dimers) are essential. Finally, the Spectrin cytoskeleton is required to regulate the localisation of the Hippo pathway effector YAP in response to cell density human epithelial cells. Our findings identify both apical and basolateral Spectrins as regulators of Hippo signalling and suggest Spectrins as potential mechanosensors.

## Introduction

The Hippo pathway transduces signals from the cell surface to the nucleus to control tissue growth and regeneration in animals (Pan, 2010; Halder & Johnson, 2011; Tapon & Harvey, 2012). Recent work implicates the Hippo pathway as a key regulator of stem cell proliferation (Camargo *et al*, 2007; Cai *et al*, 2010; Karpowicz *et al*, 2010; Shaw *et al*, 2010; Staley & Irvine, 2010; Zhang *et al*, 2010, 2011; Cordenonsi *et al*, 2011). The core of the pathway was discovered in *Drosophila* and includes the upstream kinase Hippo (MST1/2 in mammals) and the downstream kinase Warts (LATS1/2 in mammals), which acts to phosphorylate and inhibit the transcriptional activator Yorkie (Yki; YAP/TAZ in mammals) (Harvey *et al*, 2003; Udan *et al*, 2003; Wu *et al*, 2003; Huang *et al*, 2005). Yki then acts with the Mask (MASK1/2 in mammals) co-factor to switch the nuclear DNA-binding protein Scalloped (TEAD1-4 in mammals) from a transcriptional repressor to an activator (Wu *et al*, 2008; Koontz *et al*, 2013; Sansores-Garcia *et al*, 2013; Sidor *et al*, 2013).

Several proteins can act upstream of the core kinase cascade, including the apical FERM-domain proteins Expanded (Ex; similar to both FRMD6 and AMOT proteins in humans) and Merlin (Mer; the NF2 tumour suppressor in humans), which act in parallel with activate Hippo signalling (Hamaratoglu *et al*, 2006; Irvine, 2012). In *Drosophila* wing or eye epithelia, mutation of *ex* is sufficient to cause mild tissue overgrowth, but *ex, mer* double mutants cause a much stronger overgrowth phenotype, similar to *hpo* or *wts* mutants (Hamaratoglu *et al*, 2006). Ex is recruited to the membrane by the transmembrane protein Crumbs (Crb), an apical polarity determinant that can form apical cell–cell junctions in epithelia (Chen *et al*, 2010; Ling *et al*, 2010; Robinson *et al*, 2010). Mutants in *crb* therefore cause a mild overgrowth phenotype in wing and eye epithelia (Chen *et al*, 2010; Ling *et al*, 2010). Mer is also recruited to apical cell–cell junctions, where it binds to the Kibra (Kib) protein (Baumgartner *et al*, 2010; Genevet *et al*, 2010; Yu *et al*, 2010). *ex, kib* or *kib, mer* double mutants cause a strong *hpo-*like overgrowth phenotype, suggesting that the three proteins act together upstream of the core kinase cascade (Baumgartner *et al*, 2010; Genevet *et al*, 2010; Yu *et al*, 2010). In addition, *ex, kib* double mutants strongly affect polarisation of Crb in the ovarian follicular epithelium and polarisation of the actin cytoskeleton for border cell migration, functions that are independent of nuclear signalling via Yki (Fletcher *et al*, 2012; Lucas *et al*, 2013).

In mammalian cells in culture, the Hippo pathway responds to mechanical stimulation, being activated in densely confluent epithelial cells (such that YAP is cytoplasmic) and becoming inactivated when cells are sparse and stretched out across their substrate (such that YAP becomes nuclear) (Dupont *et al*, 2011; Aragona *et al*, 2013). A functioning F-actin cytoskeleton is required for this response to mechanical stimulation, but whether F-actin itself is the molecular mechanosensor or whether other molecules might mediate this function remains unclear (Dupont *et al*, 2011; Aragona *et al*, 2013). Interestingly, regulation of YAP by mechanical stretching and the F-actin cytoskeleton appears to be partly independent of LATS phosphorylation of YAP and likely involves a yet unidentified mechanism (Dupont *et al*, 2011; Aragona *et al*, 2013; Gaspar & Tapon, 2014).

Here, we identify the Spectrin cytoskeleton as crucial upstream regulator of Yki in the developing wing, eye, follicular epithelium and border cells. We further show that human Spectrins are essential for regulation of YAP in response to cell density in human cells.

## Results

**Figure 1:**
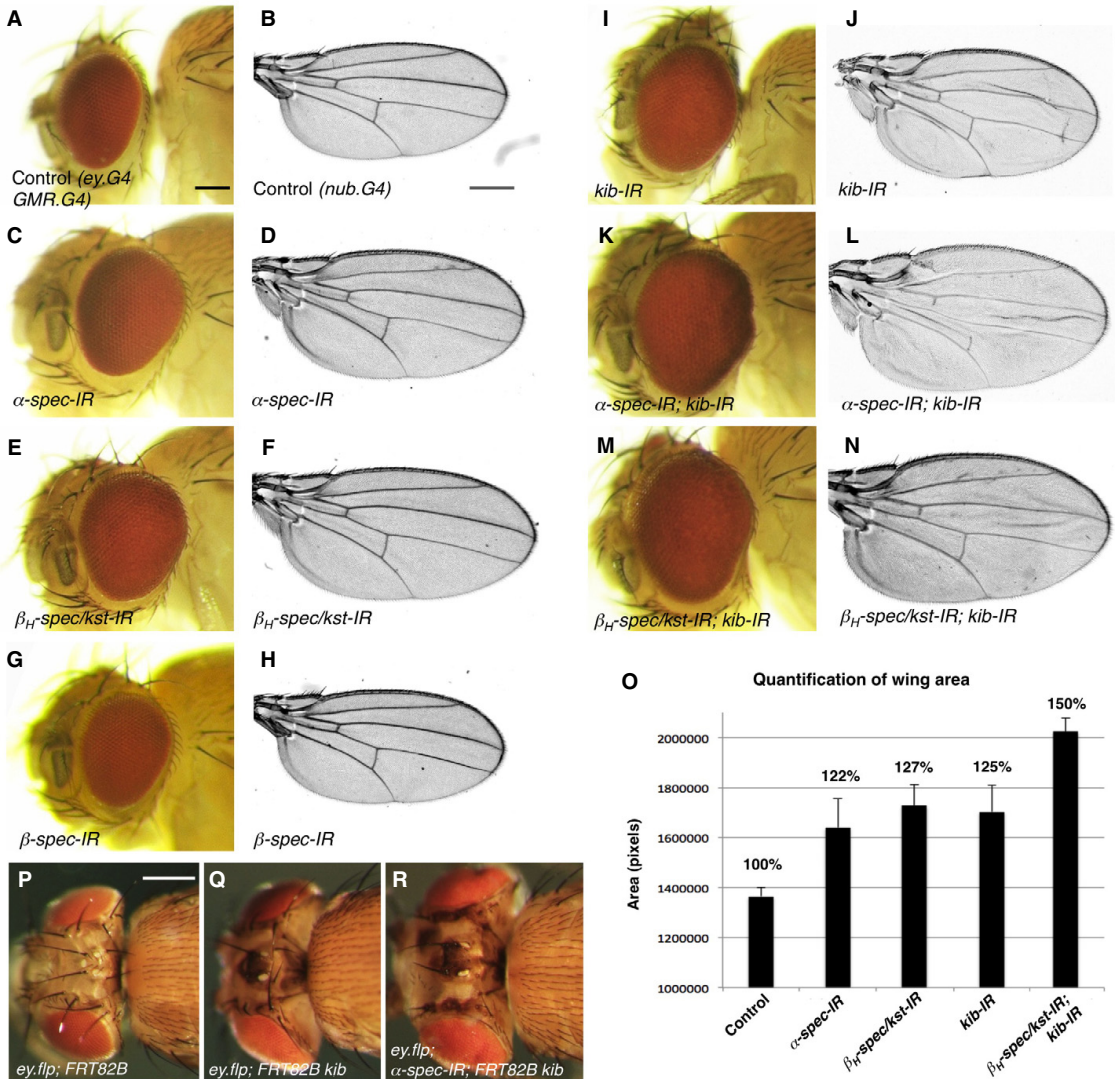
The Spectrin cytoskeleton restricts tissue growth in the *Drosophila* eye and wing

To identify novel regulators of Hippo signalling, we performed an *in vivo* RNAi screen in the *Drosophila* wing for novel genes controlling tissue growth (M. Campos & B. J. Thompson, manuscript in preparation). In this screen, we identified the apical Spectrin cytoskeleton components α-Spectrin (α-Spec) and β-heavy Spectrin (β_H_Spec)—also known as Karst (Kst)—as producing moderate wing and eye overgrowth phenotypes, similar to RNAi knock-down of Crb (Fig1A–F and Supplementary Figs S1 and S2). Spectrins are large cytoskeletal proteins that form hexagonal networks at the intracellular surface of the plasma membrane in all animal cells and have been reported to have mechanosensory properties (Bennett & Baines, 2001; Johnson *et al*, 2007; Bennett & Healy, 2009; Stabach *et al*, 2009; Meng & Sachs, 2012; Krieg *et al*, 2014). The Spectrin cytoskeleton is polarised in *Drosophila* epithelia, with dimers of α- and β_H_-Spec/Kst localising to the apical domain and dimers of α- and β-Spec localising to the basolateral domain (Thomas & Kiehart, 1994; Lee *et al*, 1997; Thomas *et al*, 1998; Thomas & Williams, 1999; Zarnescu & Thomas, 1999; Medina *et al*, 2002). Notably, RNAi knock-down of the basolateral β-Spec did not have a consistent effect on eye or wing size (Fig1G and H and Supplementary Figs S3 and S4). Furthermore, just as *crb* and *ex* mutants are known to genetically interact with *kib*, knock-down of α-Spec or β_H_-Spec/Kst strongly enhanced the overgrowth caused by a *kib* null mutant in the eye (Fig1I–R).

A-O UAS.RNAi lines were driven with *eyeless.Gal4 gmr.Gal4* for expression during eye development or *nubbin-Gal4* for expression during wing development. (A, B) Control adult *Drosophila* eye (A) and wing (B). (C, D) *α-spectrin* RNAi results in overgrowth of the eye (C) and wing (D). (E, F) *β*_*H*_*-spectrin/karst* RNAi results in overgrowth of the eye (E) and wing (F). (G, H) *β-spectrin* RNAi does not affect eye size (G) or wing size (H). (I, J) *kibra* RNAi results in overgrowth of the eye (I) and wing (J). (K, L) *α-spectrin, kibra* double RNAi results in stronger overgrowth of the eye (K) and wing (L). (M, N) *β*_*H*_*-spectrin/karst, kibra* double RNAi results in stronger overgrowth of the eye (M) and wing (N). (O) Quantification of female wing sizes by pixel area, 5 wings per genotype were measured. Error bars show standard deviation.

P-RThe eyeless FLP MARCM system was used to generate clonally mutant fly eyes. *kibra* mutant eyes (Q) overgrow slightly compared to controls (P), while *kibra* mutant eyes expressing *α-spectrin* RNAi (R) overgrow strongly compared to controls (P).

Data information: Scale bars, 250 μm.

**Figure 2:**
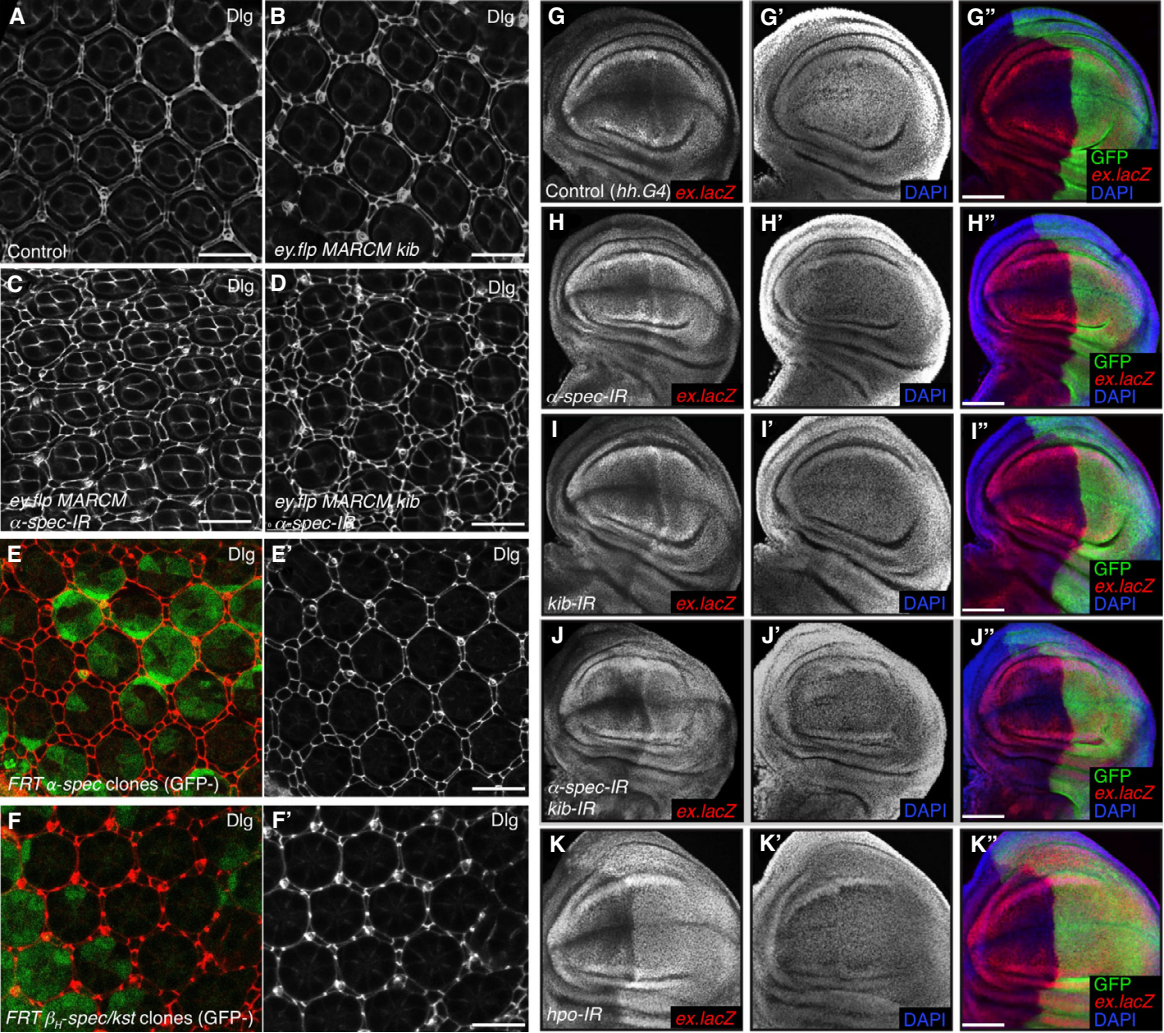
The Spectrin cytoskeleton represses interommatidial cell number and Hippo target gene expression in parallel with Kibra

Despite previous reports that apical β_H_-Spec/Kst interacts physically with Crb, genetic analysis of *β*_*H*_*-spec/kst* mutants indicated that it is dispensable for polarisation of Crb and for epithelial polarity in general (Thomas *et al*, 1998; Zarnescu & Thomas, 1999; Medina *et al*, 2002; Pellikka *et al*, 2002). Since Crb is known to regulate Hippo signalling (Chen *et al*, 2010; Ling *et al*, 2010; Robinson *et al*, 2010), we tested whether loss of apical Spectrins also affects Hippo signalling outputs. We examined interommatidial cells in the pupal retina and found that loss of α-Spec or β_H_-Spec/Kst increases cell number and this effect is magnified by concominant mutation of *kib* (Fig2A–F). We also examined the expression of the key Hippo reporter gene, *ex.lacZ*, in wing discs expressing *α-spec* RNAi in the posterior compartment with *hh.Gal4*. We found that, compared to controls, wing discs expressing *α-spec* RNAi exhibit a slightly elevated level of *ex.lacZ* expression in the posterior compartment (Fig2G and H). This elevation of *ex.lacZ* expression is similar in magnitude to that caused by *kib* RNAi and becomes stronger in *α-spec, kib* double RNAi wing discs, similar to *hpo* RNAi (Fig2I–K). These results show that apical Spectrins regulate Yki activity in the *Drosophila* wing and eye. They also show that Spectrins act in parallel with Kibra, in the same manner as Ex (Baumgartner *et al*, 2010), as the double-mutant *spectrin kibra* or *expanded kibra* each cause a stronger phenotype than the single mutants alone (Baumgartner *et al*, 2010).

A-F The eyeless FLP MARCM system was used to generate mutant eyes that also express UAS.RNAi targeting the Spectrin cytoskeleton. Pupal retinas were examined at 42–46 h after puparium formation (APF). Cell membranes were marked with Dlg staining. (A) Control pupal retina showing cone cells surrounded by interommatidial cells. (B) *kibra* mutant pupal retina displaying additional interommatidial cells. (C) Pupal retina expressing *α-spectrin* RNAi showing additional interommatidial cells. (D) *kibra* mutant pupal retinas expressing *α-spectrin* RNAi showing many additional interommatidial cells. (E, F) Clones of *α-spectrin* mutant cells (GFP negative) (E) and *β*_*H*_*-spectrin/karst* mutant cells (GFP negative) (F) show extra interommatidial cells. Scale bars, 20 μm.

G-K UAS.RNAi lines were driven with *hh.Gal4 UAS.GFP* for expression in the posterior compartment and contained the *ex.lacZ* reporter transgene. (G) Control wing showing a normal *ex.lacZ* expression pattern, which is low at the dorsal–ventral boundary but high in the proximal regions of the wing disc. (H) RNAi knock-down of *α-Spectrin* results in a mild up-regulation of *ex.lacZ* in the posterior compartment. (I) RNAi knock-down of *kibra* results in a mild up-regulation of *ex.lacZ* in the posterior compartment. (J) Dual RNAi knock-down of both *α-spectrin* and *kibra* results in enhanced up-regulation of *ex.lacZ* in the posterior compartment. (K) RNAi knock-down of *hippo* results in an up-regulation of *ex.lacZ* in the posterior compartment. Scale bars, 50 μm.

**Figure 3:**
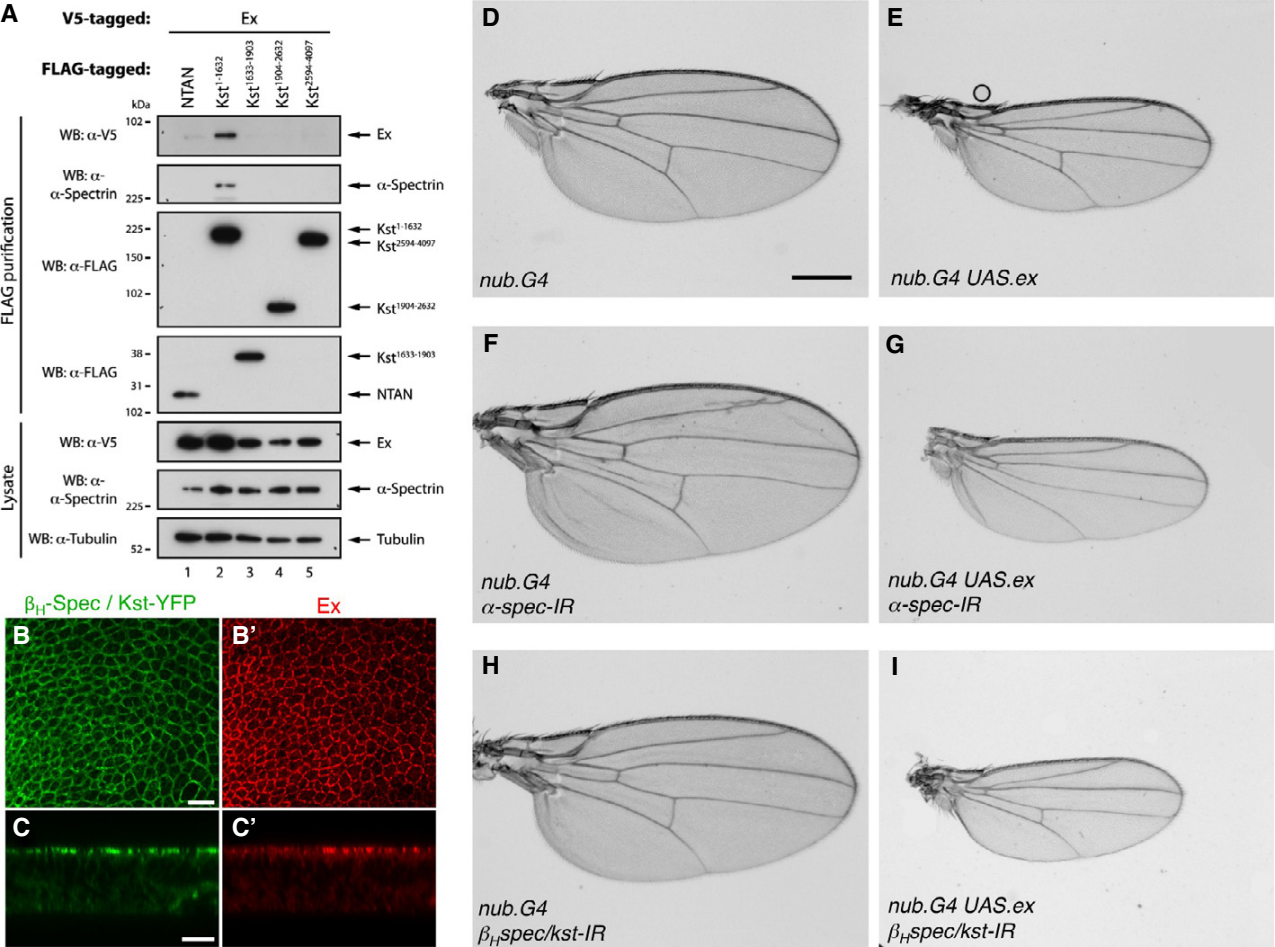
The apical α-β_H_ Spectrin cytoskeleton binds to and co-localises with Expanded protein and acts genetically upstream or at the level of Expanded to control wing growth

We next investigated the mechanism by which apical Spectrins regulate Yki activity. Since Spectrins act in parallel with Kibra—similar to Ex (Baumgartner *et al*, 2010)—and have been shown to physically associate with Crb (Medina *et al*, 2002; Pellikka *et al*, 2002)—again similar to Ex (Chen *et al*, 2010; Ling *et al*, 2010; Robinson *et al*, 2010)—we tested whether Ex might interact with Spectrins. We performed co-immunoprecipitation experiments from *Drosophila* S2 cells expressing V5-tagged Ex and a series of constructs expressing portions of the very large β_H_-Spec/Kst protein. We found that Ex interacts strongly with the N-terminal region of β_H_-Spec/Kst (Fig3A). Pulling down the N-terminal region of β_H_-Spec/Kst with Ex also co-immunoprecipitated endogenous α-Spec, which is known to form dimers with β_H_-Spec/Kst (Fig3A). *In vivo*, we found that β_H_-Spec/Kst co-localises with Ex at the apical domain of wing disc epithelial cells (Fig3B and C). α-Spec or β_H_-Spec/Kst is not required to localise Ex or Crb apically (Supplementary Fig S5 and data not shown). Instead, they appear to be required for normal activation of Ex signalling to Hpo and Wts, as the tissue overgrowth phenotype of α-Spec or β_H_-Spec/Kst knock-down is completely suppressed by overexpression of Ex (Fig3D–I). These results show that apical Spectrins bind to and co-localise with Ex and act genetically upstream or at the level of Ex to regulate signalling to Yki.

A Co-IP of V5-tagged Expanded with the FLAG-tagged N-terminal region of Karst/β_H_-Spectrin, but not other Karst truncation constructs.

B, C Expanded co-localises with Karst at the apical membrane of wing imaginal disc cells (apical view, B; cross section, C).

D Control nubbin.Gal4 expressing wing.

E Overexpression of Ex activates Hippo signalling to produce a small wing.

F RNAi knock-down of *α-Spectrin* results in an overgrown wing.

G Overexpression of Ex blocks the effect of *α-Spectrin* RNAi.

H RNAi knock-down of *β*_*H*_*-spectrin/karst* results in an overgrown wing.

I Overexpression of Ex blocks the effect of *β*_*H*_*-spectrin/karst* RNAi.

Data information: Scale bars, 10 μm (B, C), 250 μm (D–I).

**Figure 4:**
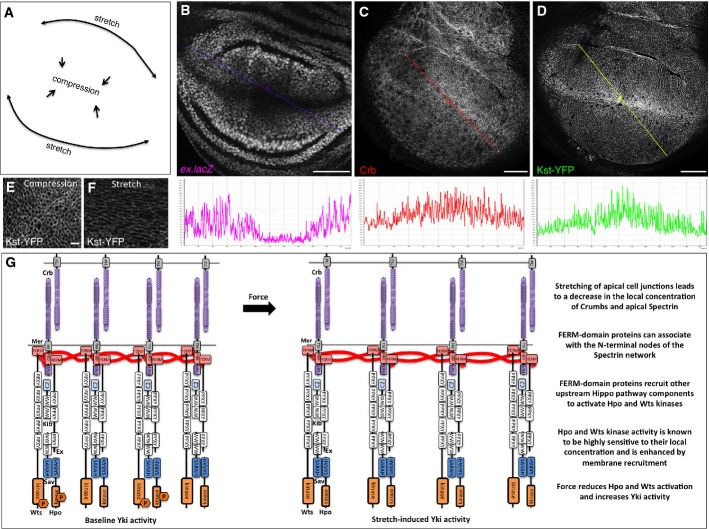
The apical α-β_H_ Spectrin cytoskeleton may be mechanosensory in the wing imaginal disc

Hippo signalling has been proposed to have a possible mechanosensory role in the *Drosophila* wing disc, where a pattern of stretching and compression of cells at their apical surfaces correlates with the pattern of Yki activity as measured with *ex.lacZ* (Fig4A and B; Aegerter-Wilmsen *et al*, 2007; Legoff *et al*, 2013; Mao *et al*, 2013; Schluck *et al*, 2013). We found that this pattern of stretching and compression influences the intensity of Crb and apical Spectrin staining in cells (Fig4A–F). Thus, Crb and β_H_-Spec/Kst are concentrated at the junctions of small compressed cells and diluted at the junctions of stretched cells in a manner that inversely correlates with *ex.lacZ* expression. This correlation suggests a potential model of mechanosensory regulation of Yki activity via Spectrin-dependent clustering of Crb complexes (Fig4G). According to this model, stretching of cells would exert force upon the apical Spectrin cytoskeleton that would de-cluster Crb complexes and therefore reduce Hpo and Wts activation and increase Yki activity (Fig4G; see also Discussion).

A Schematic diagram of central compression and circumferencial stretching in the third instar wing pouch.

B-D Third instar wing imaginal disc stained for beta-galactosidase expressed from the expanded.lacZ reporter gene (B), Crb (C) and Kst-YFP (D). Quantification of line intensity shown below. Note inverse correlation of Crb and Kst-YFP with *ex.lacZ*. Scale bars, 50 μm.

E, F Zoom of Kst-YFP cells under compression (E) and under stretch (F). Scale bars, 10 μm.

G Schematic diagram of the effect of force on the apical spectrin cytoskeleton leading to declustering of Crumbs complexes and reduced Hippo signalling.

**Figure 5:**
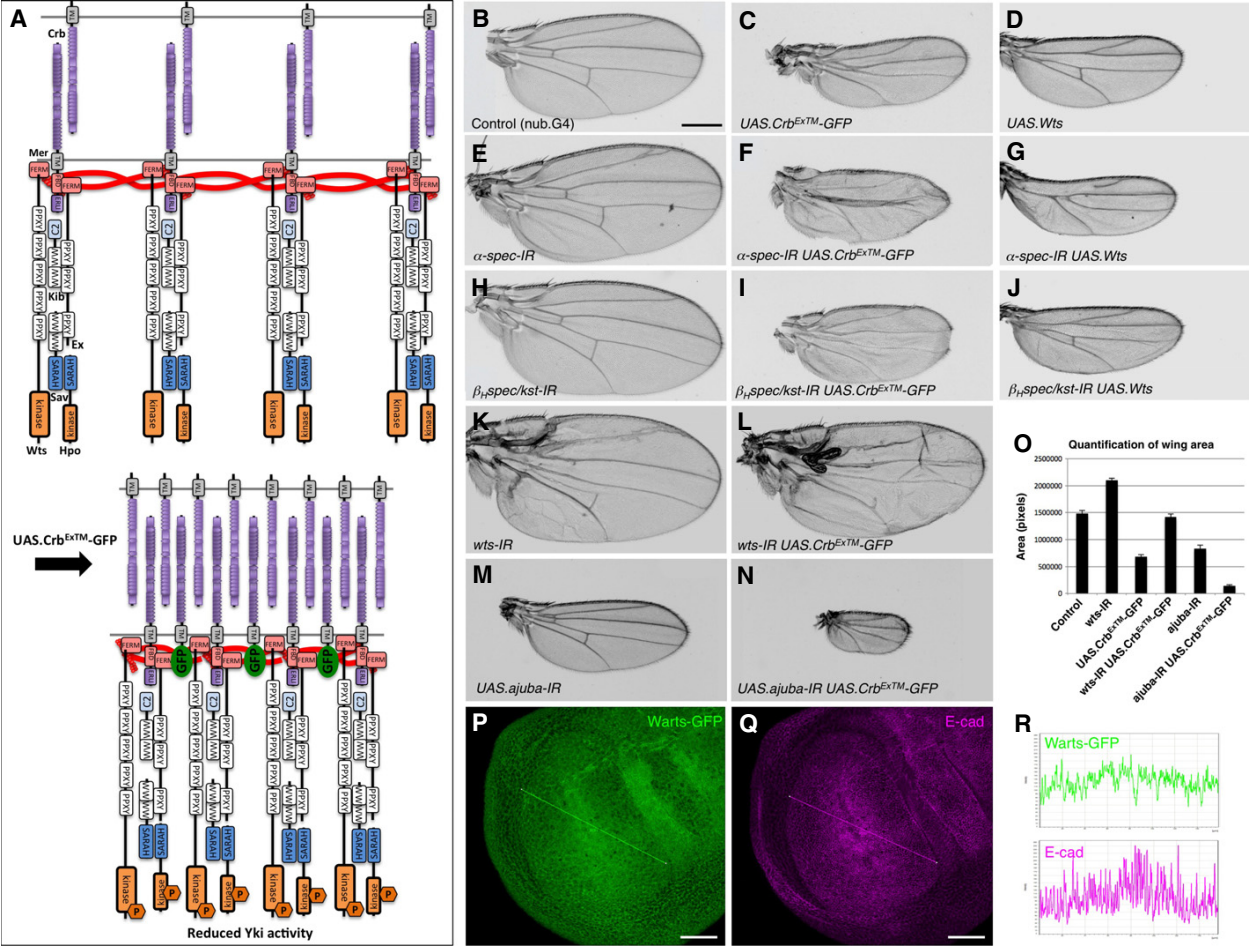
Crb^Ex^™-GFP restricts tissue growth and acts upstream of Wts and in parallel with Ajuba

To test this model, we aimed to induce clustering of Crb complexes by overexpression of a form of Crb whose intracellular domain was replaced with GFP (Crb^ExTM^-GFP; Fig5A) (Pellikka *et al*, 2002; Thompson *et al*, 2013). Overexpression of Crb^ExTM^-GFP during wing development with nub.Gal4 resulted in a small wing phenotype, highly similar to overexpression of Wts (Fig5B–D). Furthermore, the overgrowth phenotype caused by RNAi knock-down of apical Spectrins was completely suppressed by co-expression of either Crb^ExTM^-GFP or Wts (Fig5E–J). Crb^ExTM^-GFP appears to act upstream of Wts, because the tissue undergrowth phenotype induced by Crb^ExTM^-GFP is suppressed by RNAi knock-down of Wts (Fig5K and L). These results are consistent with the notion that clustering of Crb complexes induces Hippo signalling to inhibit tissue growth, lending support to a model of mechanosensation involving clustering of Crb complexes. However, other interpretations and models of mechanosensation are also possible.

A Schematic diagram of clustering of Crb complexes by expression of Crb^ExTM^-GFP.

B Control *nub.Gal4* wing.

C, D Expression of *UAS.Crb*^*ExTM*^*-GFP* with *nub.Gal4* (C) or *UAS.Wts* with *nub.Gal4* (D) results in a small wing.

E RNAi knock-down of *α-Spectrin* results in an overgrown wing.

F, G Expression of *UAS.Crb*^*ExTM*^*-GFP* (F) or *UAS.Wts* (G) blocks the effect of *α-Spectrin* RNAi.

H RNAi knock-down of *β*_*H*_*-spectrin/karst* results in an overgrown wing.

I, J Expression of *UAS.Crb*^*ExTM*^*-GFP* (I) or *UAS.Wts* (J) blocks the effect of *α-Spectrin* RNAi.

K, L RNAi knock-down of Wts results in a strongly overgrown wing (K) and expression of *UAS.Crb*^*ExTM*^*-GFP* does not block the effect of *wts* RNAi (L).

M, N RNAi knock-down of Ajuba results in a small wing (M), and expression of *UAS.Crb*^*ExTM*^*-GFP* enhances the effect of *ajuba* RNAi (N).

O Quantification of various wing sizes. Error bars show standard deviation.

P, Q Localisation of Wts-GFP (P) and E-cadherin (Q) in the third instar wing imaginal disc.

R Quantification of line intensity in (P) and (Q). No increased Wts-GFP intensity is observed in proximal regions where cells are subject to stretching.

Data information: Scale bars, 250 μm (B–N), 40 μm (P, Q).

One alternative model of mechanosensation involves recruitment of Warts to E-cadherin via the Ajuba protein (Rauskolb *et al*, 2014). This mechanism appears to be distinct from and to act in parallel with the Spectrin/Crumbs-mediated version we propose, because loss of Ajuba and overexpression of Crb^ExTM^-GFP have an additive effect in suppressing tissue growth (Fig5M–O). Furthermore, we do not observe increased recruitment of Warts-GFP to E-cadherin in response to tissue stretching in the wing imaginal disc (Fig5P–R), suggesting that this alternative model does not explain the physiological control of Hippo signalling in this context. A second alternative model of mechanosensation involves activation of the JNK pathway by forces (Codelia *et al*, 2014). However, blocking JNK signalling in *Drosophila* does not affect tissue growth, and there is no evidence for physiological regulation of JNK activation by forces in the wing imaginal disc. A third alternative model of mechanosensation involves the actin cytoskeleton, which can directly influence the nuclear localisation of the Yki homologues YAP and TAZ in mammalian cell culture independently of MST and LATS kinases (Dupont *et al*, 2011; Aragona *et al*, 2013). Current evidence suggests that all effects of the actin cytoskeleton on Yki activity appear to be mediated via Hpo and Wts (Sansores-Garcia *et al*, 2011), although further work is needed to rigorously test whether Wts-independent regulation of Yki also occurs in *Drosophila* (Gaspar & Tapon, 2014). Thus, we currently favour the view that apical Spectrins act with Crb complexes to help sense forces by activating Hpo-Wts signalling during *Drosophila* wing and eye development.

We next tested whether loss of Spectrins can produce an *ex-*like phenotype in other tissue contexts. In the ovarian follicular epithelium, Ex is known to act in parallel with Kibra to regulate polarisation of Crb, such that *ex, kib* double-mutant cells accumulate Crb in vesicles (Fletcher *et al*, 2012). This role of Ex and Kibra does not require more downstream signalling components such as *wts* (Fletcher *et al*, 2012). We found that β_H_-Spec/Kst co-localises with Ex at the apical domain of follicle cells, while Kibra is present both apically and in the cytoplasm in a punctate pattern that co-localises with the Exocyst complex—with which Kibra has been shown to interact (Rosse *et al*, 2009) (Supplementary Fig S6A–C). We found that loss of apical Spectrins alone does not affect Crb, but loss of apical Spectrins in combination with mutation of *kib* or *sec15* (encoding an Exocyst component) causes a strong accumulation of Crb in vesicles (Supplementary Fig S6D–M). Thus, apical Spectrins are involved in Ex-mediated regulation of Crb polarisation in follicle cells.

**Figure 6:**
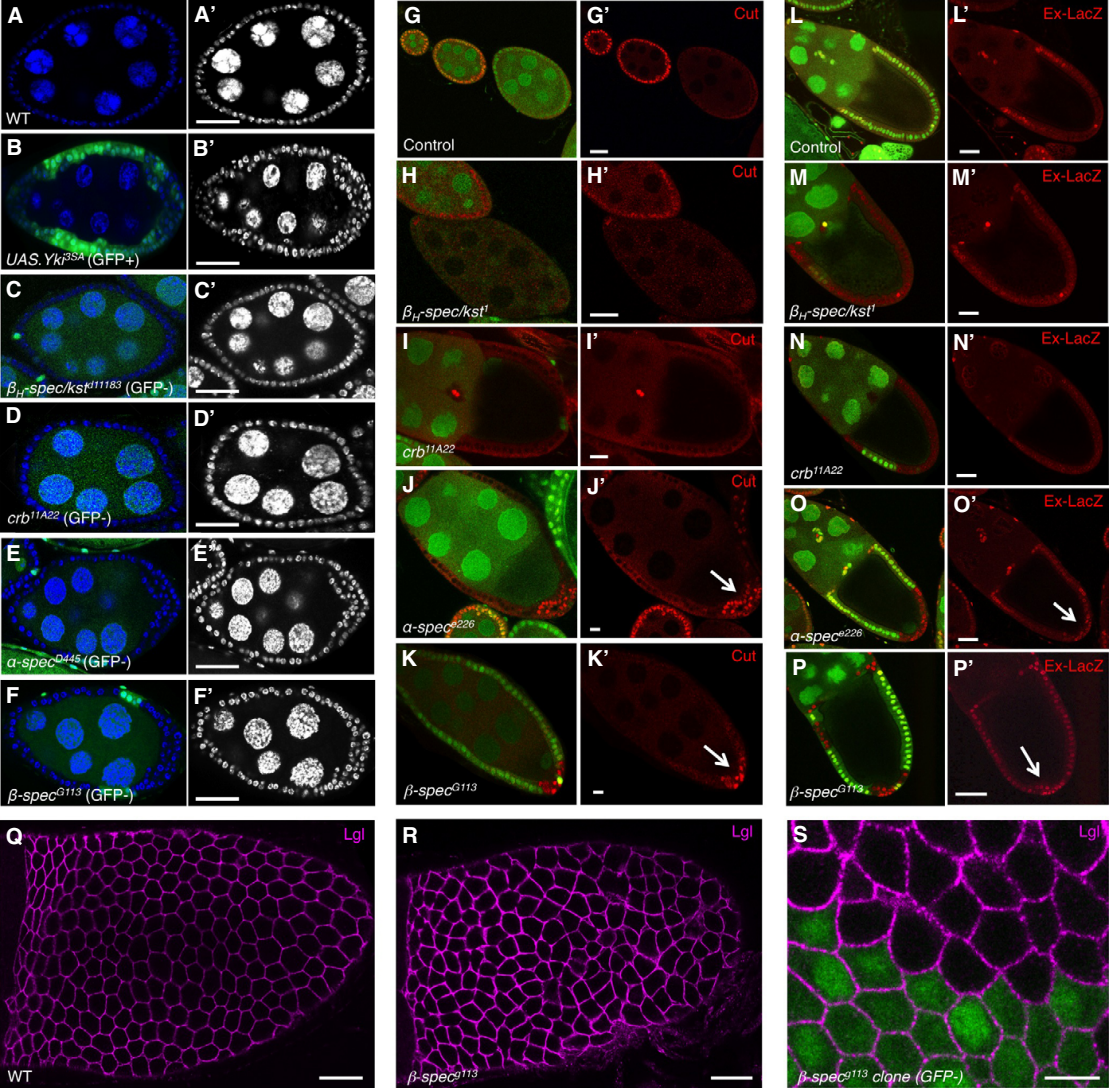
Basolateral *α*-*β* Spectrins are required for Hippo signalling and membrane tension in the *Drosophila* follicular epithelium

We noted that *α-spec* RNAi in *kib* mutant clones caused a strong overproliferation and multilayering phenotype in follicle cells (Supplementary Fig S6M). We therefore tested whether Spectrins are required for regulation of Yki activity in the ovarian follicular epithelium. Yki is known to promote early proliferation and suppress apoptosis in follicle cells until stage 6, during which time it expresses the transcription factor Cut (Huang & Kalderon, 2014). After stage 6, Cut expression is silenced and cells arrest proliferation and switch to an endocycle. When Hippo signalling is disrupted after stage 6, Cut is known to be re-expressed in a group of posterior follicle cells that continue to proliferate (Meignin *et al*, 2007; Polesello & Tapon, 2007; Genevet *et al*, 2010; Yu *et al*, 2010). Surprisingly, we found that mutation of either *β*_*H*_*-spec/kst* or *crb* is not sufficient to drive early overproliferation (Fig6A–D) or to induce Cut in late-stage posterior follicle cells (Fig6G–I). Multilayering only occurs in around 30% of *crb* mutant clones, while mutation of *α-spec* or *β-spec* leads to early overproliferation and multilayering in 95% of clones (Fig6E and F) and also leads to re-expression of Cut in the posterior follicle cells (Fig6J and K). Furthermore, another Hippo target gene, *ex.lacZ*, is also ectopically expressed in posterior follicle cells mutant for *α-spec* or *β-spec* but not in *β*_*H*_*-spec/kst* or *crb* mutant clones (Fig6L–P). Notably, mutation of *β-spec* causes a disruption of membrane tension such that the normally regular hexagonal shape of follicle cells becomes disorganised (Fig6Q–S). These findings indicate that the basolateral α-β Spectrin cytoskeleton controls membrane tension and Hippo signalling to the nucleus in the follicular epithelium.

A Control egg chamber stained for DAPI to mark nuclei.

B Overexpression of Yki3SA stimulates follicle cell proliferation (MARCM clone, GFP positive).

C-F Mutation of *β*_*H*_*-spectrin/karst* (C) or *crb* (D) does not increase follicle cell proliferation (GFP negative clone), while mutation of *α-spectrin* (E) or *β-spectrin* (F) stimulates follicle cell proliferation (GFP negative clone).

G Control egg chamber stained for Cut, a marker of proliferating cells that is normally down-regulated after stage 6 of oogenesis.

H-K Mutation of *β*_*H*_*-spectrin/karst* (H) or *crb* (I) does not increase Cut expression in follicle cells (GFP negative clone), while mutation of *α-spectrin* (J) or *β-spectrin* (K) stimulates Cut expression in posterior follicle cells (GFP negative clone).

L Control egg chamber showing *ex.lacZ* reporter gene expression.

M-P Mutation of *β*_*H*_*-spectrin/karst* (M) or *crb* (N) does not increase *ex.lacZ* expression in follicle cells (GFP negative clone), while mutation of *α-spectrin* (O) or *β-spectrin* (P) stimulates *ex.lacZ* expression in posterior follicle cells (GFP negative clone).

Q Wild-type follicle cells showing normal hexagonal packing of epithelial membranes.

R Mutation of *β-spectrin* disrupts the normal hexagonal packing, suggesting a role in maintaining membrane tension.

S Clone of *β-spectrin* mutant cells (GFP negative) showing differential membrane tension at the interface with wild-type cells (GFP positive).

Data information: Scale bars, 25 μm (A–R), 10 μm (S).

A small group of anterior follicle cells, called border cells, are known to delaminate from the epithelium at stage 8 of development and migrate across the egg chamber to reach the oocyte at the posterior by stage 10. We previously showed that upstream Hippo pathway components have a key role in promoting border cell migration by regulating the actin cytoskeleton (Lucas *et al*, 2013). We found that *α-spec* RNAi knock-down in *kib* mutant border cell clusters causes a strong delay in reaching the oocyte by stage 10 of oogenesis (Supplementary Fig S7A–E). *α-spec* RNAi knock-down alone, or mutation of *kib* alone, causes a milder phenotype (Supplementary Fig S7E). Interestingly, RNAi knock-down of *β*_*H*_*-spec/kst* did not cause a phenotype, alone or in combination with *kib* (Supplementary Fig S7E). Accordingly, we found that β_H_-Spec/Kst is not actually expressed in border cells, while β-Spec localises around the entire plasma membrane with α-Spec (Supplementary Fig S7F–I). These results indicate that α-β Spectrin dimers regulate Hippo signalling during border cell migration.

**Figure 7:**
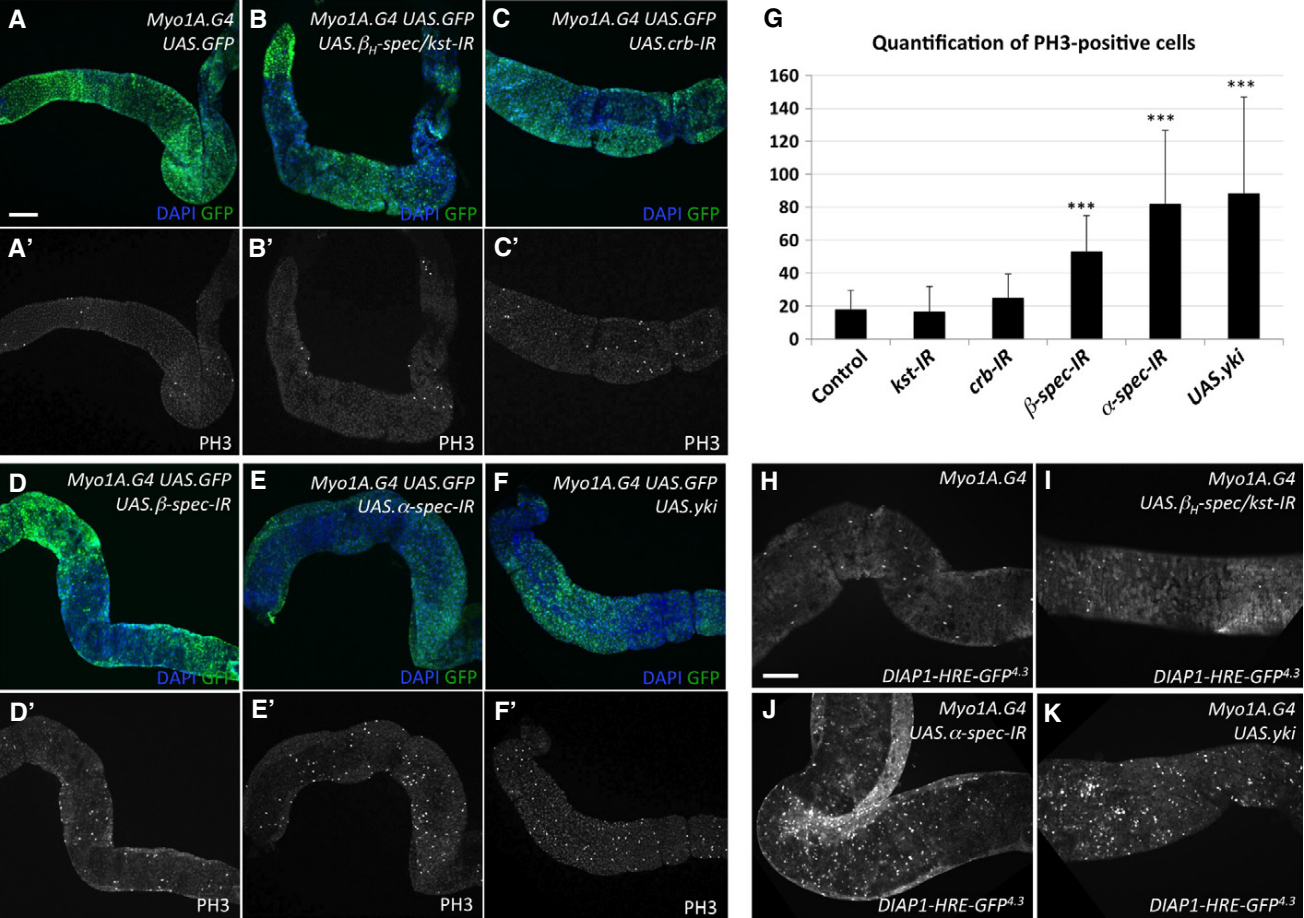
Basolateral *α*-*β* Spectrins are required to restrict cell proliferation in the *Drosophila* intestinal epithelium

The Hippo pathway was recently shown to have a key role in regulating stem cell proliferation in the *Drosophila* intestine (Karpowicz *et al*, 2010; Shaw *et al*, 2010; Staley & Irvine, 2010). RNAi knock-down of *wts* or overexpression of *yki* in enterocytes with the *myo1A.G4* driver leads to induction of stem cell proliferation (Karpowicz *et al*, 2010; Shaw *et al*, 2010; Staley & Irvine, 2010). However, the upstream regulators that control Hippo signalling in the intestine have not been identified. We therefore examined the requirement for Crb and Spectrins in the adult fly intestine. We found that RNAi of *crb* or apical *β*_*H*_*-spec/kst* did not lead to stem cell overproliferation (Fig7A–C). However, RNAi silencing of *α-spec* or *β-spec* does produce a strong stem cell proliferation response, similar to overexpression of Yki (Fig7D–G). Furthermore, a Hippo pathway reporter gene *DIAP1-HRE-GFP* is activated in the overproliferating stem cells (Fig7H–K). These results indicate that the basolateral α-β Spectrin cytoskeleton, rather than the apical α-β_H_-Spectrin cytoskeleton, is crucial to promote Yki activity in enterocytes.

A Control *myo1A.Gal4 UAS.GFP* adult midgut stained for phospho-histone H3 to mark mitotic stem cells.

B-E RNAi knock-down of *β*_*H*_*-spectrin/karst* (B) or *crumbs* (C) does not increase stem cell proliferation, while RNAi knock-down of *β-spectrin* (D) or *α-spectrin* (E) strongly stimulates stem cell proliferation.

F Overexpression of Yki strongly stimulates stem cell proliferation.

G Quantification of the number of PH3-positive mitotic cells. *N* > 13 samples. Statistical analysis performed using the two-tailed Student's *t*-test. Error bars show standard deviation. ***P-value ≤ 0.001.

H-K A Hippo reporter DIAP1-HRE-GFP is normally expressed in sporadic stem cells. Its expression is not affected by RNAi knock-down of *β*_*H*_*-spectrin/karst* (I), but is up-regulated in many stem cells by RNAi knock-down of *α-spectrin* (J) or by overexpression of Yki (K).

Data information: Scale bars, 100 μm.

**Figure 8:**
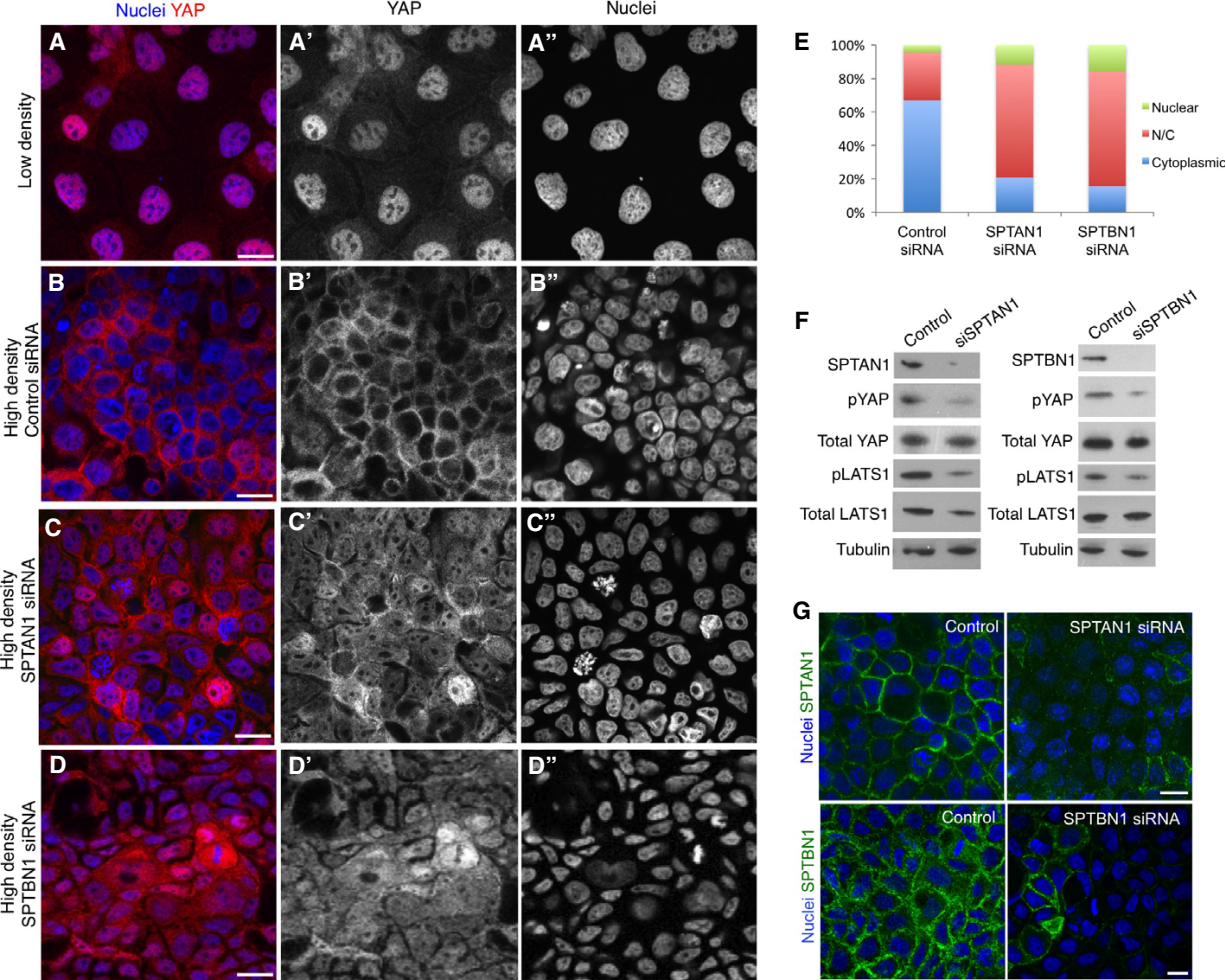
Human Spectrin SPTAN1 and SPTBN1 are required for the YAP localisation response to cell density

The Spectrin cytoskeleton is conserved between *Drosophila* and humans, but is more complex in humans as it features many variant β-Spectrins. To test whether the Spectrin cytoskeleton also regulates Hippo signalling in human cells, we silenced expression of the sole human non-erythrocyte alpha-Spectrin protein, SPTAN1, and a major non-erythrocyte β-Spectrin, SPTBN1, by siRNA transfection of Caco-2 cells. The human homologue of Yki, called YAP, is normally nuclear in sparsely plated cells, but becomes cytoplasmic in densely confluent epithelial monolayers due to mechanical cues (Dupont *et al*, 2011) (Fig8A and B). We found that siRNA knock-down of SPTAN1 or SPTBN1 in densely confluent epithelia is sufficient to send YAP to the nucleus and to reduce YAP phosphorylation and LATS1 phosphorylation (Fig8C–G). These results show that the Spectrin cytoskeleton is crucial for regulation of YAP in response to density in human cell culture and that disruption of Spectrins has similar effects to mechanical stretching of cells by plating at low density.

A Control Caco-2 epithelial cells plated a low density show strong nuclear YAP localisation.

B Control Caco-2 epithelial cells plated at high density show relocalisation of YAP to the cytoplasm.

C, D siRNA knock-down of SPTAN1 (C) or SPTBN1 (D) prevents relocalisation of YAP to the cytoplasm.

E Quantification of YAP localisation in (B–D).

F Western blotting reveals that SPTAN1 or SPTBN1 are required for normal levels of YAP phosphorylation, and of LATS1 phosphorylation.

G siRNAs against SPTAN1 or SPTBN1 efficiently deplete their target proteins.

Data information: Scale bars, 20 μm.

## Discussion

Our results identify the Spectrin cytoskeleton as an important upstream regulator of the Hippo signalling pathway in both *Drosophila* and human cells. In all fly tissues we have examined, Spectrins are essential to maintain full repression of Yki activity. However, different tissues exhibit different dependence on apical α-β_H_ versus basolateral α-β Spectrins. The localisation of Spectrin proteins in each model tissue we have examined is summarised in Supplementary Fig S8.

In the developing wing and eye, apical α-β_H_-Spectrins act with Crb and Ex to activate Hippo signalling and thereby restrict tissue growth (Figs1 and 2). Since Crb binds to Ex (Chen *et al*, 2010; Ling *et al*, 2010), and Ex can bind to both Kibra (Genevet *et al*, 2010) and Spectrins (Fig2), and Crb can also associate with Spectrins (Medina *et al*, 2002; Pellikka *et al*, 2002), these five proteins are likely to form a complex at the apical domain of *Drosophila* epithelial cells. Kibra is also able to associate with the FERM-domain protein Merlin (Mer) at the apical domain (Genevet *et al*, 2010; Yu *et al*, 2010), and we are able to co-immunoprecipitate both Kibra and Mer with apical α-β_H_ Spectrins (Supplementary Fig S9). In contrast, Hpo and Wts localise primarily to the cytoplasm, but must transiently associate with upstream pathway components at the plasma membrane in order to be activated (Lucas *et al*, 2013; Yin *et al*, 2013). Direct binding of Wts to Mer and indirect binding of Hpo via Sav to a network of WW domain–PPXY motif interactions appear to facilitate activation of Hpo and Wts at the plasma membrane, where molecular clustering may induce Hpo auto-phosphorylation and phosphorylation of Wts to drive signalling. In the wing and eye epithelium, apical α-β_H_ Spectrins may assist this signal-activating process by forming a dense meshwork that promotes clustering of Crb-Ex-Kib-Mer complexes to induce Hpo and Wts kinase activity to restrict growth (Fig4). In support of this model, inducing clustering of endogenous Crb by overexpressing a form of Crb lacking the intracellular domain is sufficient to overcome the loss of Spectrins and to suppress wing growth (Fig5).

In the developing follicular epithelium and adult intestinal epithelium, basolaterally localised α-β Spectrins, rather than apical α-β_H_ Spectrins, are crucial for repression of Yki activity (Figs6 and 7). This finding may explain why apical Crb is surprisingly not required for Yki repression in these tissues (Figs6 and 7). This finding is all the more remarkable because apical α-β_H_ Spectrins do act in parallel with Kibra to polarise Crb in follicle cells (Supplementary Fig S6). Thus, the mere presence of Crb, Ex, Kib and α-β_H_ Spectrins in a cell does not necessitate that these proteins will form the primary control mechanism for the regulation of Yki. How basolateral α-β Spectrins connect to Hippo signalling and/or regulation of Yki in these tissues remains to be explored. Nevertheless, in all *Drosophila* tissues we have examined, either the α-β or α-β_H_ Spectrins are necessary for normal repression of Yki.

In human epithelial cells, polarisation of α (SPTAN1) and β (SPTBN1) Spectrins is much less apparent than in *Drosophila* (Supplementary Fig S8). Nevertheless, our data show that these Spectrins are essential for regulating the classic YAP localisation response to cell density in culture (Fig8), as are homologues of Crb (CRB3), Ex (AMOT) and Mer (NF2) (Zhao *et al*, 2007, 2011; Varelas *et al*, 2010). This response to cell density was shown to be a mechanosensory response that requires an intact F-actin cytoskeleton (Dupont *et al*, 2011). However, the identity of the mechanosensory molecule(s) that mediate this response is not clear. Our findings suggest that the Spectrin cytoskeleton is a candidate for this Hippo pathway mechanosensor.

Several lines of evidence support a mechanosensory function for Spectrins. Firstly, electron microscopy studies show that Spectrins form a dense meshwork at the plasma membrane that can stretch out under force, separating the N-terminal nodes at which FERM-domain proteins bind (Hirokawa *et al*, 1983; Byers & Branton, 1985; Ursitti *et al*, 1991). Secondly, individual Spectrin molecules can deform under force via conformational change (Johnson *et al*, 2007; Stabach *et al*, 2009). Thirdly, construction of a FRET-based Spectrin sensor reveals that Spectrins are under tension and can sense force in human cells in culture (Meng & Sachs, 2012) and in *C. elegans* neurons (Krieg *et al*, 2014). Finally, loss of Spectrins causes a loss of membrane tension in *C. elegans* neurons (Krieg *et al*, 2014) as well as red blood cells (Stokke *et al*, 1986), and our results are consistent with a similar role in *Drosophila* epithelial cells (Figs2A–F and 6Q–S). Thus, it is likely that force may cause alterations in the Spectrin cytoskeleton that modulate Hippo signalling, possibly by spatially separating the N-terminal nodes of the network that bind to FERM-domain proteins, thereby inducing de-clustering of upstream pathway components and signal inhibition (Fig4G).

Since Spectrins can bind to F-actin, it is also possible that force upon Spectrins may influence the actin cytoskeleton and therefore potentially influence Yki activation independently of Hpo and Wts activation, as has been shown for the mammalian homologues YAP/TAZ (Dupont *et al*, 2011; Aragona *et al*, 2013). Furthermore, Wts has also been shown to influence F-actin polymerisation via regulation of Ena/VASP proteins (Lucas *et al*, 2013), raising the possibility that canonical Hpo-Wts signalling may also act via the regulation of F-actin dynamics to control Yki, in addition to direct phosphorylation of the Yki protein by Wts. Further work is necessary to test whether direct F-actin regulation of Yki localisation occurs in *Drosophila* as it does in mammalian cells (Gaspar & Tapon, 2014).

Regardless of which signalling mechanisms Spectrins use to control Yki activity, their molecular nature makes them excellent candidate mechanosensors and Spectrins are clearly able to influence Yki-dependent tissue growth. Furthermore, there is good evidence that forces can and do contribute to promoting cell proliferation in *Drosophila* wing epithelia (Aegerter-Wilmsen *et al*, 2007; Legoff *et al*, 2013; Mao *et al*, 2013; Schluck *et al*, 2013), and it has been proposed that forces may also drive proliferation in the follicular epithelium (Wang & Riechmann, 2007). In addition, input from the Dachsous-Fat pathway to Hippo signalling is mediated via the atypical myosin Dachs, which has been shown to promote tension at apical cell–cell junctions (Mao *et al*, 2011). Thus, it is not implausible that forces could be sensed by *Drosophila* Spectrins as part of the physiological control of tissue growth during development and that this function could help explain how disparate upstream regulators of Hippo signalling, such as Crb-Crb junctions and the Dachsous-Fat cadherin system, converge to control Yki activity.

A physiological role for Spectrins as sensors of tension that regulate Hippo signalling may well be conserved across metazoa. For example, the dramatic effect of forces on human skin growth is well known, and the Hippo pathway has been shown to regulate skin proliferation in mice (Schlegelmilch *et al*, 2011; Silvis *et al*, 2011; Zhang *et al*, 2011). Notably, a recently proposed alternative mechanism for mechanosensation involving recruitment of Wts to α-catenin via the Ajuba protein is less likely to be conserved in mammals (Das Thakur *et al*, 2010; Rauskolb *et al*, 2014). In *ajuba* mutants, Wts recruitment to α-catenin is disrupted, leading to stronger Hippo signalling and tissue undergrowth in *Drosophila* (Rauskolb *et al*, 2014). However, in mammals, loss of α-catenin leads to weaker Hippo signalling and tissue overgrowth in mouse skin (Schlegelmilch *et al*, 2011; Silvis *et al*, 2011) and loss of adherens junctions also weakens Hippo signalling to activate YAP in cultured human cells (Kim *et al*, 2011). Thus, our identification of Spectrins as potential mechanosensors in the Hippo pathway provides an important novel mechanism that is more likely to be conserved in all metazoans and may be a critical control mechanism in both normal development and cancer.

## Materials and Methods

Mitotic clones in follicle cells were generated using the FLP/FRT system and were marked either negatively (absence of GFP) or positively (presence of GFP; MARCM). Third instar larvae were heat-shocked once at 37°C for 1 h and dissected 4 days after eclosion. Expression of *UAS*-driven transgenes in follicle cells was achieved with the MARCM system and in wing imaginal disc cells with the posterior compartment driver *hh.Gal4* and in enterocytes with *Myo1A.G4*.

### *Drosophila* genotypes

See Supplementary Materials and Methods for all *Drosophila* genotypes used in this study.

### Immunostaining of ovaries, imaginal discs and pupal retinas

Ovaries and imaginal discs were dissected in PBS, fixed for 20 min in 4% paraformaldehyde in PBS, washed for 30 min in PBS/0.1% Triton X-100 (PBST) and blocked for 15 min in 5% normal goat serum/PBST (PBST/NGS). Primary antibodies were diluted in PBST/NGS, and samples were incubated overnight at 4°C. Optical cross sections through the middle of egg chambers are shown in all figures.

Primary antibodies used were as follows: mouse anti-Cut (1:100 DSHB), mouse anti-βgal (Promega, 1:500), rabbit anti-expanded (1:200, gift from A. Laughon, University of Wisconsin-Madison, Madison, WI, USA), rabbit anti-PKCζ (C-20) (1:250; Santa Cruz), rat anti-Crumbs (1:300; U. Tepass), mouse anti-Dlg (1:250, DSHB), rabbit anti-Kibra (1:200; Genevet *et al*, 2010) and guinea pig anti-Sec 15 (1:1,000, gift from H. Bellen, Howard Hughes Medical Institute, Baylor College of Medicine).

Secondary antibodies (all from Molecular Probes; Invitrogen) were used at 1:500 for 2–4 h prior to multiple washes in PBST and staining with DAPI at 1 μg/ml for 10–30 min prior to mounting on slides in Vectashield (Vector labs). Images were taken with a Leica SP5 confocal microscope and processed with Adobe Photoshop.

### Immunostaining of intestinal epithelia

Flies were maintained on standard media, which was changed every 3 days. Crosses were set up and maintained at 18°C until adulthood. Three- to four-day-old adults were shifted to 29°C for 10 days for induction of transgene expression. Female adult flies were dissected in 1× PBS. The gastrointestinal tract was removed and fixed in 0.5× PBS with 4% paraformaldehyde for 30 min. Samples were washed in 0.1% Triton X-100 (PBST), permeabilised for 30 min in 0.3% PBST and pre-blocked for 1 h in 10% NGS before incubation with primary antibody overnight at 4°C in 5% NGS. Samples were washed in PBST and pre-blocked for 1 h in 10% NGS before incubation with secondary antibody in 5% NGS for 2 h at room temperature. Samples were stained with DAPI and washed in PBST, followed by PBS and mounted in Vectashield.

The following primary antibody was used: rabbit anti-phospho-histone H3 (Millipore) 1/1,000. Secondary antibodies (all from Molecular Probes; Invitrogen) were used at 1:500. Images were acquired on a Zeiss LSM710 confocal microscope and were processed using Adobe Photoshop.

### Molecular biology

The Expanded expression plasmid was generated previously (Genevet *et al*, 2010). β-heavy Spectrin expression plasmids were generated using Gateway technology. Constructs detailed below were PCR-amplified and cloned into the pDONR Zeo Entry vector. For Destination vectors, the pAWF and pAWH plasmids from the *Drosophila* Gateway Vector Collection were used.

The constructs (and primers) used were as follows:

Karst isoform A; aa 1–1632
Fwd primer—5′ atgacccagcgggacggcatcatcaag 3′
Rev primer—5′ ggactttgctgccggcaggtgttc 3′
Karst isoform A; aa 1633–1903
Fwd primer—5′ atgaatgaattgggtcagaatctgcaccaagccc 3′
Rev primer—5′ gagttgttcctgtcgcttctcaacctcatt 3′
Karst isoform A; aa 1904–2632
Fwd primer—5′ atgagaacttcctgggagaaccttctccagc 3′
Rev primer—5′ ctgctcgccctgttgtttcagtccagc 3′
Karst isoform A; aa 2594–4097
Fwd primer—5′ atgcagtcaccatccactatcaacgattgcgag 3′
Rev primer—5′ ctgtggcgggacttgactcgagcg 3′

### Co-immunoprecipitation

*Drosophila* S2 cell extracts were prepared as previously described (Genevet *et al*, 2010), and FLAG-tagged proteins were purified using anti-FLAG M2 Affinity Agarose Gel (Sigma) before elution with lysis buffer [50 mM Tris pH 7.5, 150 mM NaCl, 1% Triton X-100, 10% glycerol and 1 mM EDTA, plus phosphatase inhibitor cocktails 1 and 2 (Sigma), protease inhibitor cocktail (Roche) and 0.1 M NaF] supplemented with FLAG peptide. Detection of purified proteins and associated complexes was performed using chemiluminescence (GE Healthcare). Western blots were probed with anti-FLAG (mouse M2; Sigma), anti-V5 (mouse Novex®, Life Technologies), anti-α-Spectrin (mouse 3A9, Developmental Studies Hybridoma Bank—DHSB) and anti-Tubulin (mouse E7, DSHB) antibodies.

For Co-IP from embryos, *Drosophila* Karst YFP knock-in embryos (DGRC 115285) or Wiso embryos were collected over 24 h at 22°C before being lysed in buffer containing 10 mM Tris pH 7.5, 150 mM NaCl, 0.5% NP-40 and 0.5 mM EDTA (Chromotek), plus PhosSTOP Phosphatase Inhibitor Cocktail Tablets (Roche), protease inhibitor cocktail (Roche), 0.1 M NaF and 1 mM PMSF. Samples were left on ice to solubilise for 20 min, before being centrifuged at high speed (14,000 rpm for 30 min at 4°C) and the supernatant collected, pre-cleared and incubated with GFP Trap-M beads (Chromotek). Western blots were probed with anti-GFP (mouse; Roche), anti-Merlin (guinea pig, R. Fehon), anti-α-Spectrin (mouse 3A9, Developmental Studies Hybridoma Bank—DHSB) and anti-Kibra (rabbit, Genevet *et al*, 2010) antibodies, before being detected with chemiluminescence (GE Healthcare).

### Cell culture and transfection

Human Caco-2 adenocarcinoma colon cells were grown in DMEM (Gibco: 41966) containing l-glutamine and sodium pyruvate supplemented with 10% heat-inactivated FCS and 100 μg/ml streptomycin and 100 μg/ml penicillin. Cells were maintained in a 37°C incubator at 5% atmospheric CO_2._ All siRNA transfections were performed with interferin transfection reagent (Polyplus) using antibiotic-free media. Caco-2 cells were seeded upon 10 mm coverslips coated with 20 μg/ml fibronectin in a 48-well plate at either low density or high density, and 2 h after seeding treated with the siRNA/transfection mix. A final concentration of 100 nM siRNA was used. The following day, the media was changed and another round of siRNA transfection was performed. Cells were left for a total of 72 h before being processed for either immunofluorescence or immunoblotting. siRNA oligonucleotides used for RNAi of SPTAN1 and SPTBN1 were as a siGENOME SMARTpool (Thermo scientific).

For fixation, cells were washed with 1× PBS and either fixed in 4% paraformaldehyde in PBS for 15 min, before permeabilising with 0.3% Triton X-100 in blocking buffer (PBS containing 0.5% BSA, 10 mM glycine, and 0.1% sodium azide), or −20°C methanol for 5 min, before rehydrating with PBS. Samples were then washed and incubated in blocking buffer for 1 h before staining. Primary and secondary antibodies were incubated in blocking buffer. Coverslips were mounted using prolong anti-fade reagent (Invitrogen).

### Antibodies, image acquisition and quantification for cell culture

Antibodies used in the paper were as follows: mouse and rabbit anti-SPTAN1 (Santa Cruz and CST); mouse anti-SPTBN1 (Santa Cruz); rabbit anti-YAP (Santa Cruz), rabbit anti-pYAP (CST); rabbit anti-pLATS1 and anti-LATS1 (CST); mouse α-tubulin (Sigma). Secondary antibodies used were either from Jackson immunoresearch or from Invitrogen. DNA was stained with DAPI 1:1,000 and samples imaged with a Zeiss 710 or a Leica SP5 confocal microscope. Quantification of YAP localisation was scored in three categories: N = nuclear; N/C = nuclear and cytoplasmic and C = cytoplasmic. Cells were assessed over three independent experiments counting 350–500 cells per condition.

## Supporting Information

Supplementary Figure Legends

Review Process File
