# Supplementary figures and images for: The Spectrin cytoskeleton regulates the Hippo signalling pathway
Source: EMBO J. 2015 Feb 23;34(7):940–54. doi: 10.15252/embj.201489642 (PMC4388601; doi:10.15252/embj.201489642)

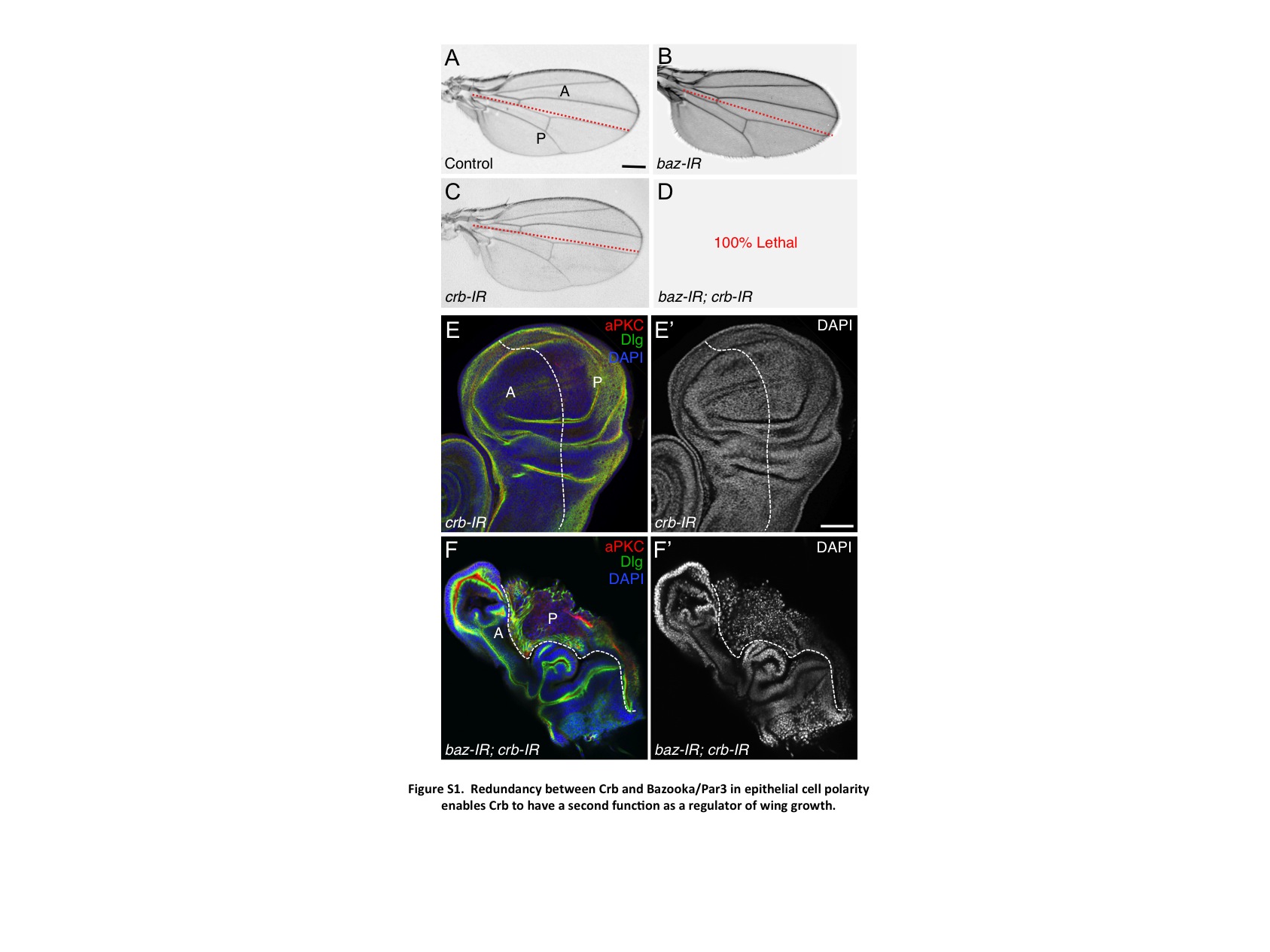

Supplement: Supplementary file 1 [file embj0034-0940-sd1.tif]

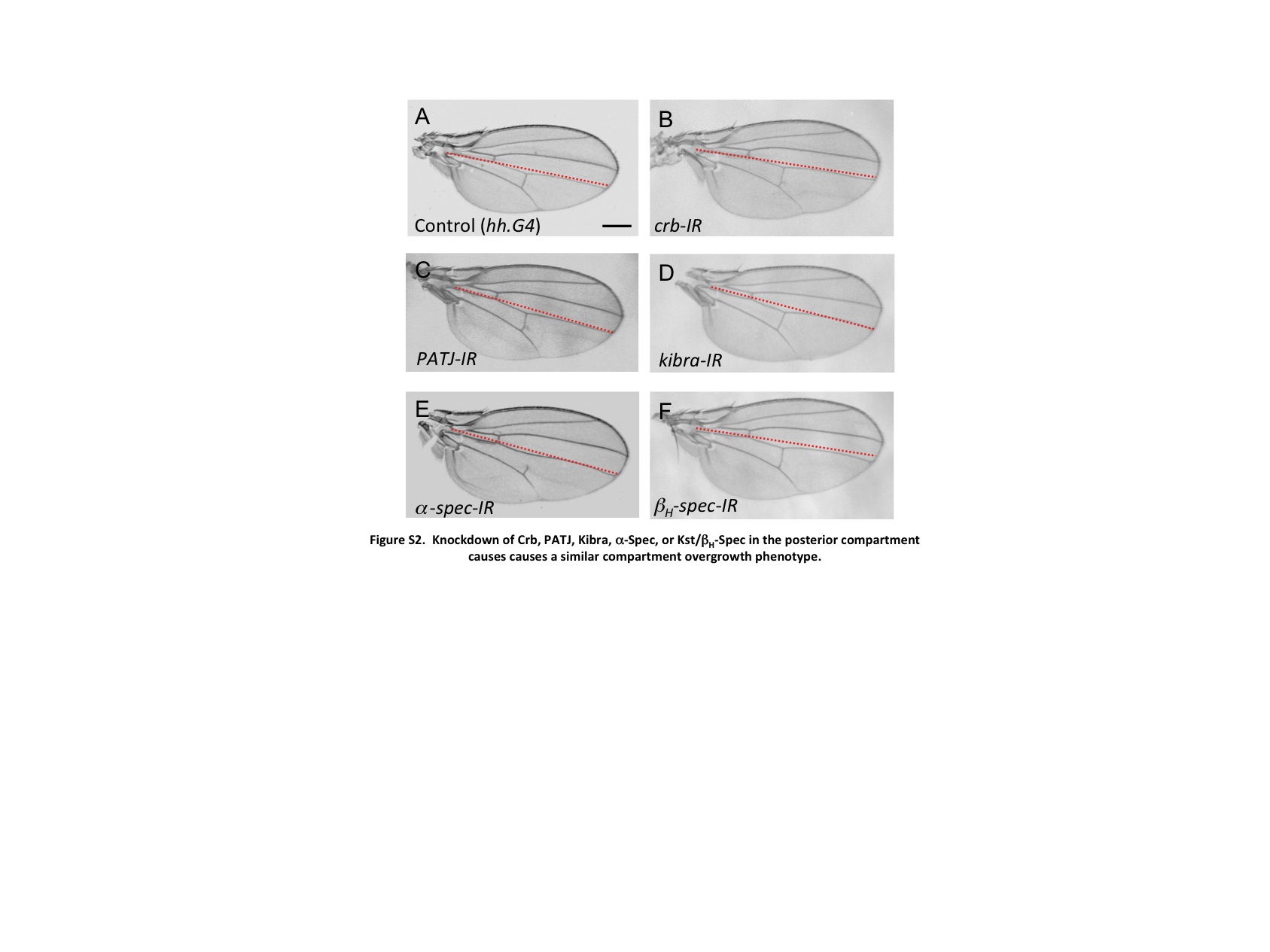

Supplement: Supplementary file 2 [file embj0034-0940-sd2.tif]

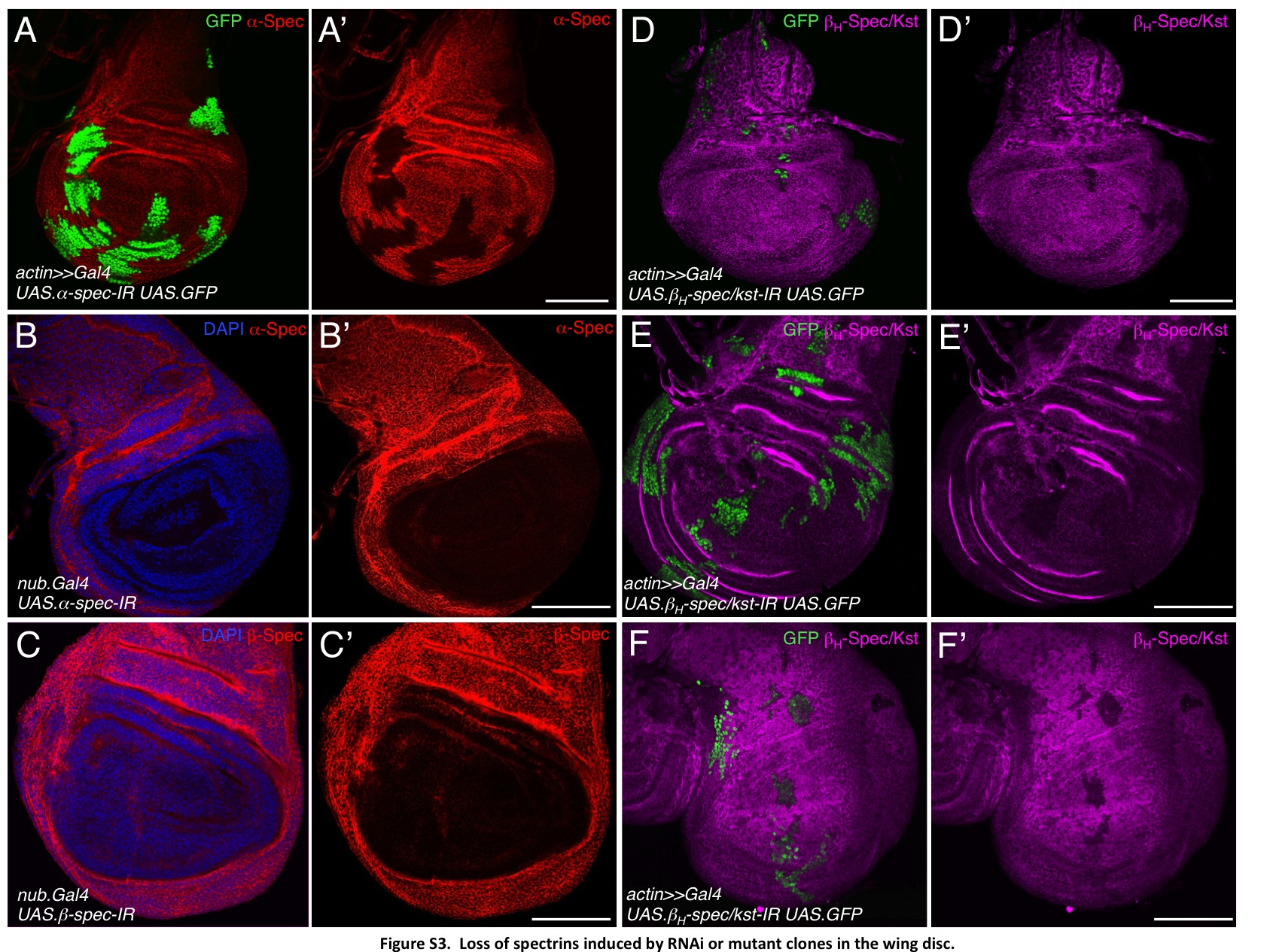

Supplement: Supplementary file 3 [file embj0034-0940-sd3.tif]

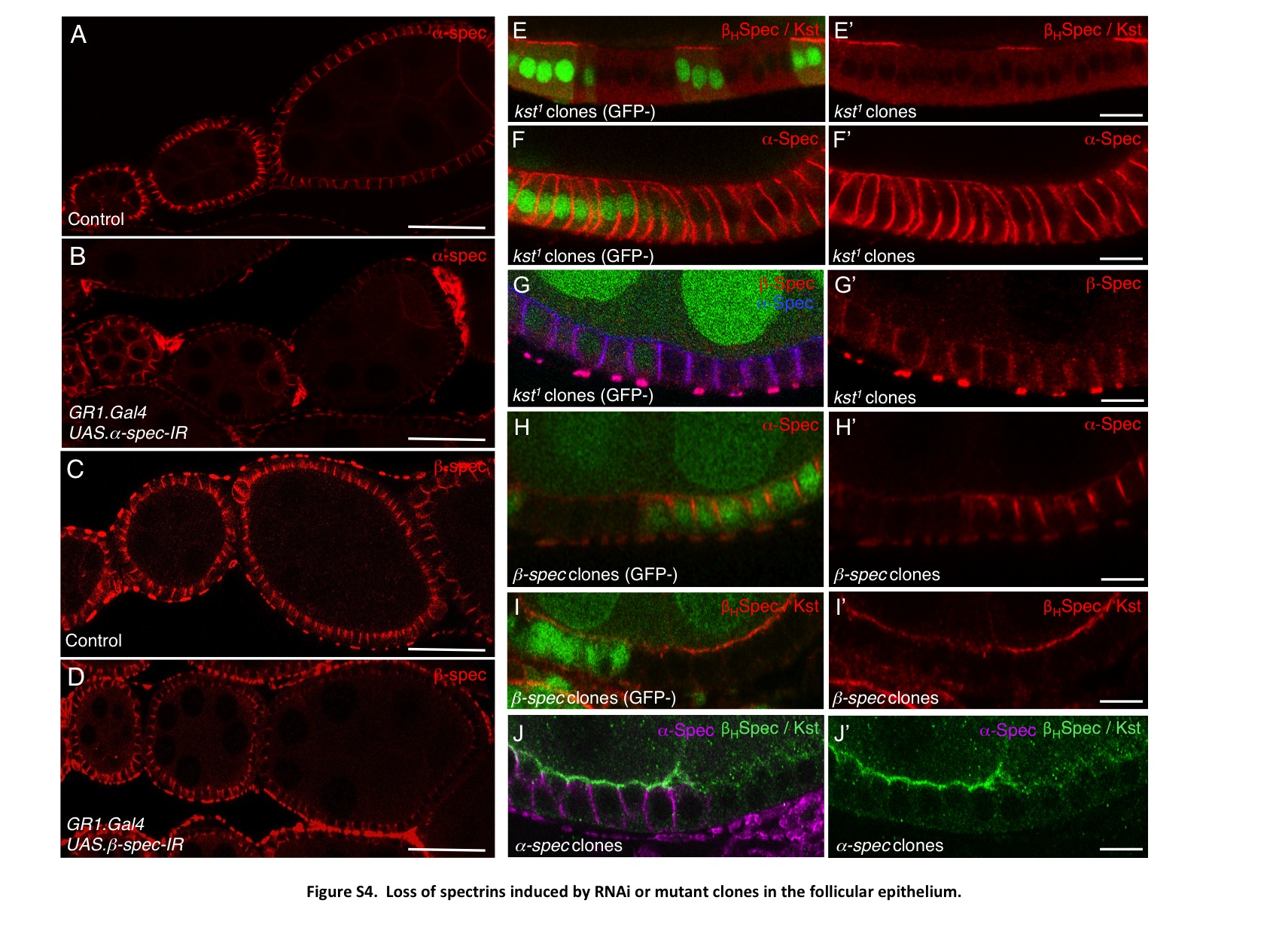

Supplement: Supplementary file 4 [file embj0034-0940-sd4.tif]

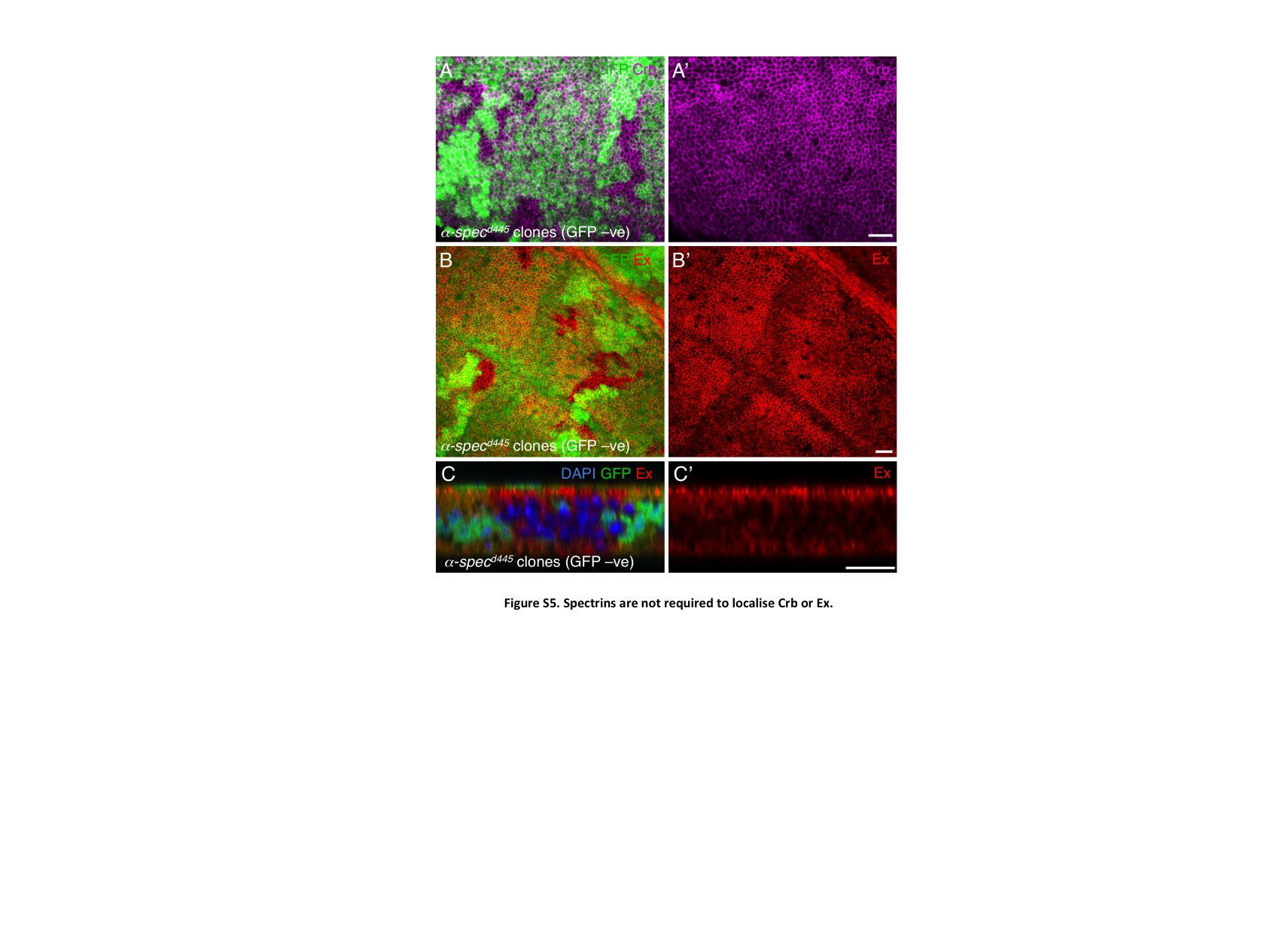

Supplement: Supplementary file 5 [file embj0034-0940-sd5.tif]

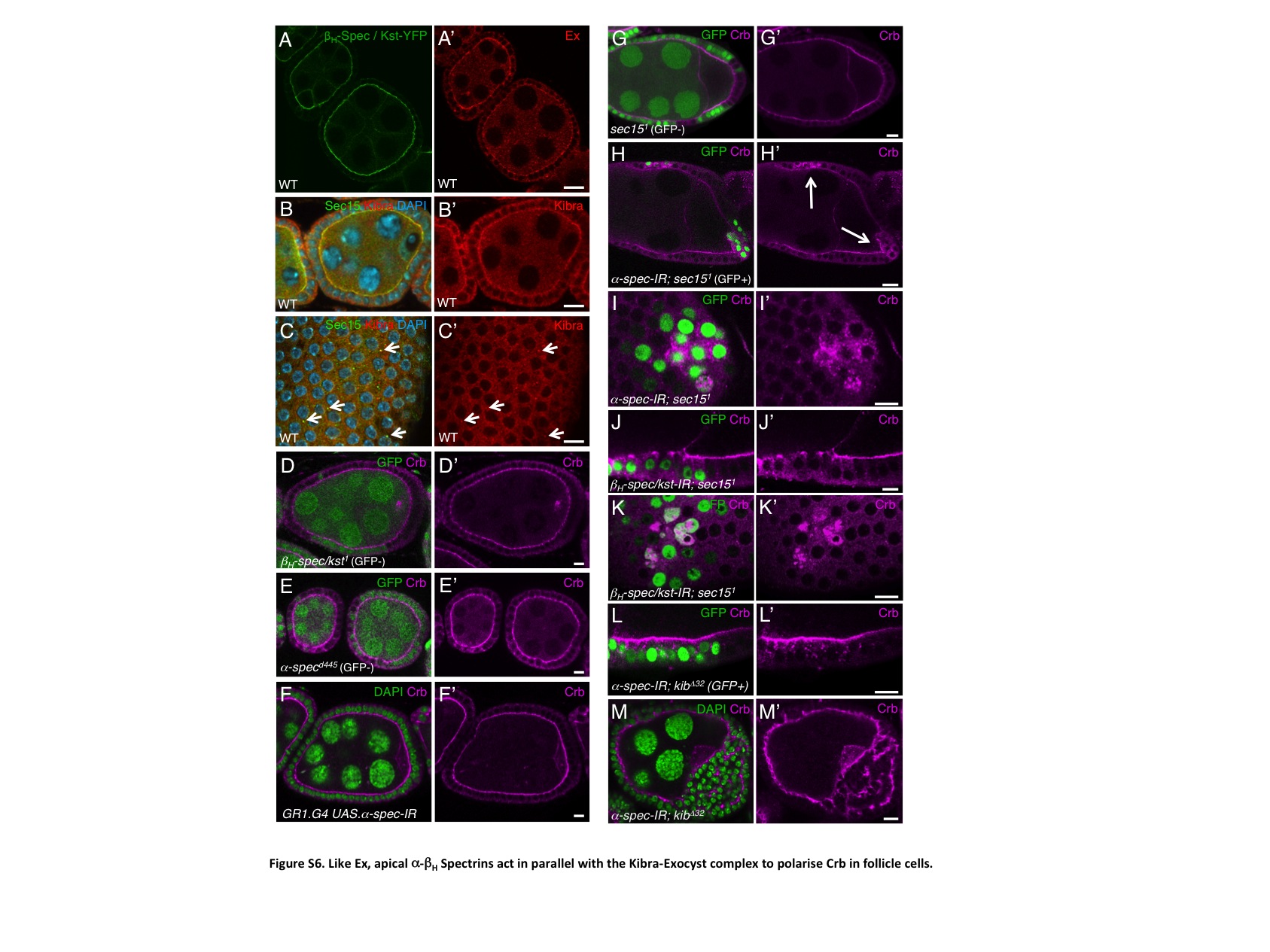

Supplement: Supplementary file 6 [file embj0034-0940-sd6.tif]

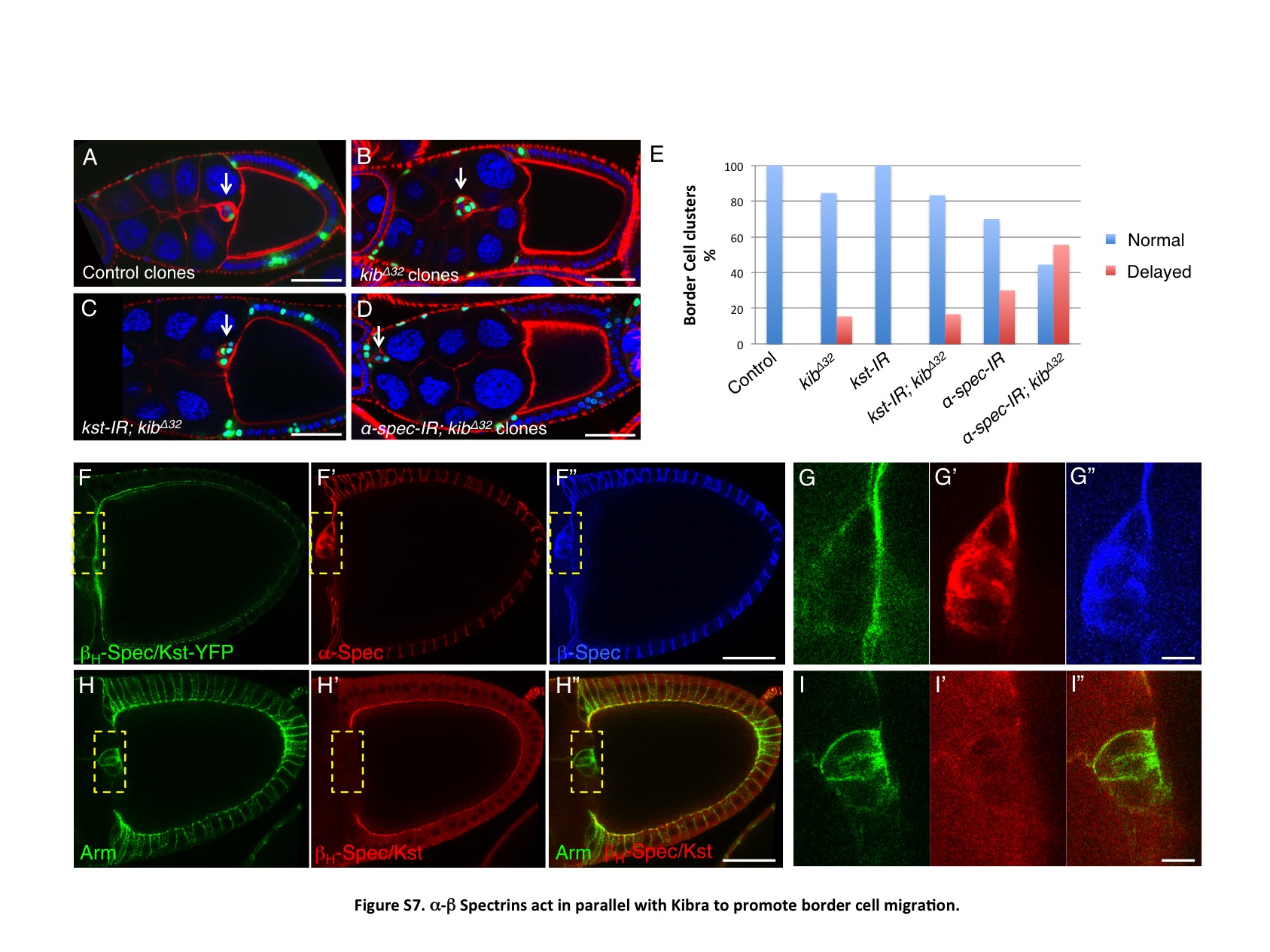

Supplement: Supplementary file 7 [file embj0034-0940-sd7.tif]

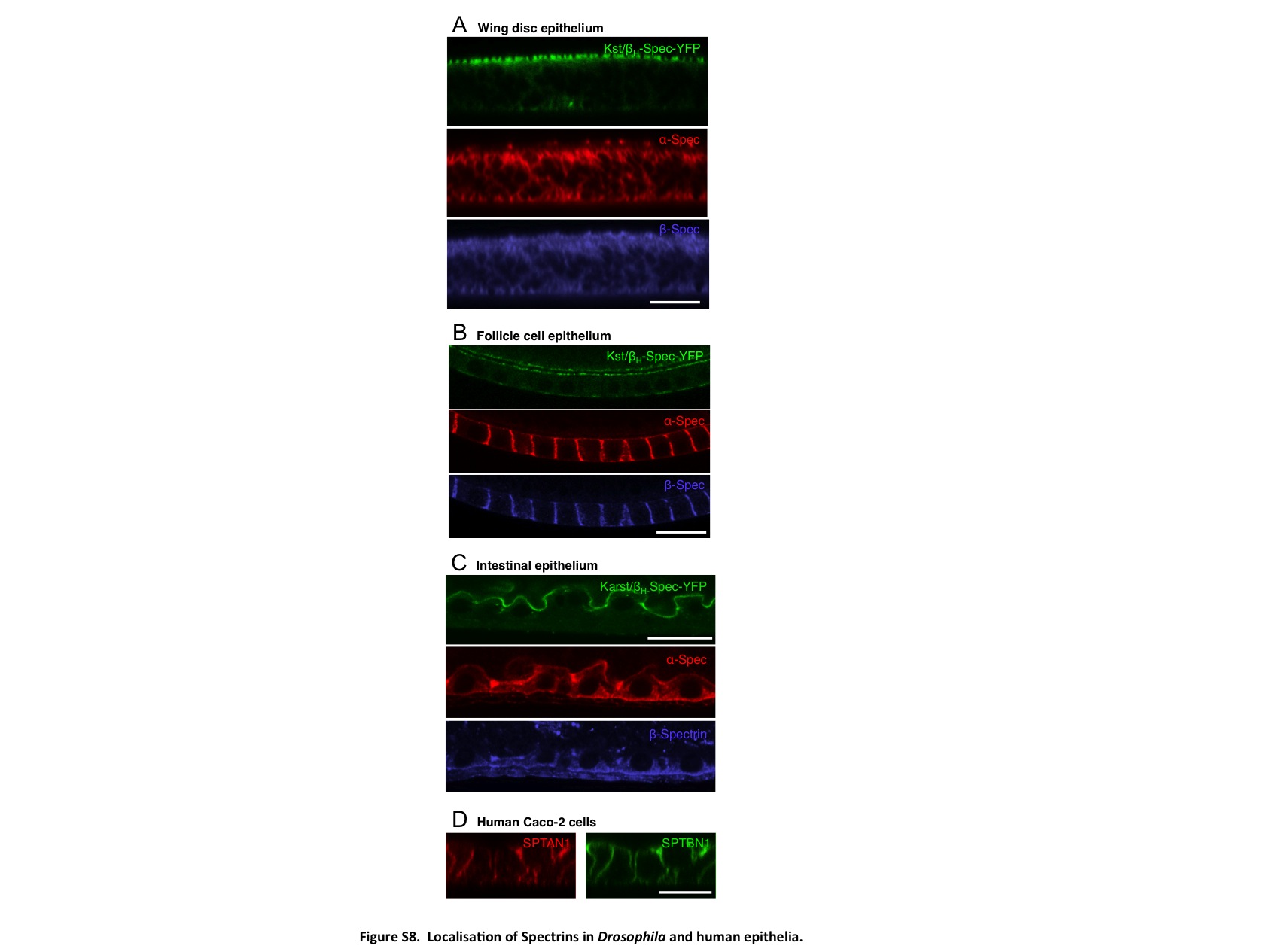

Supplement: Supplementary file 8 [file embj0034-0940-sd8.tif]

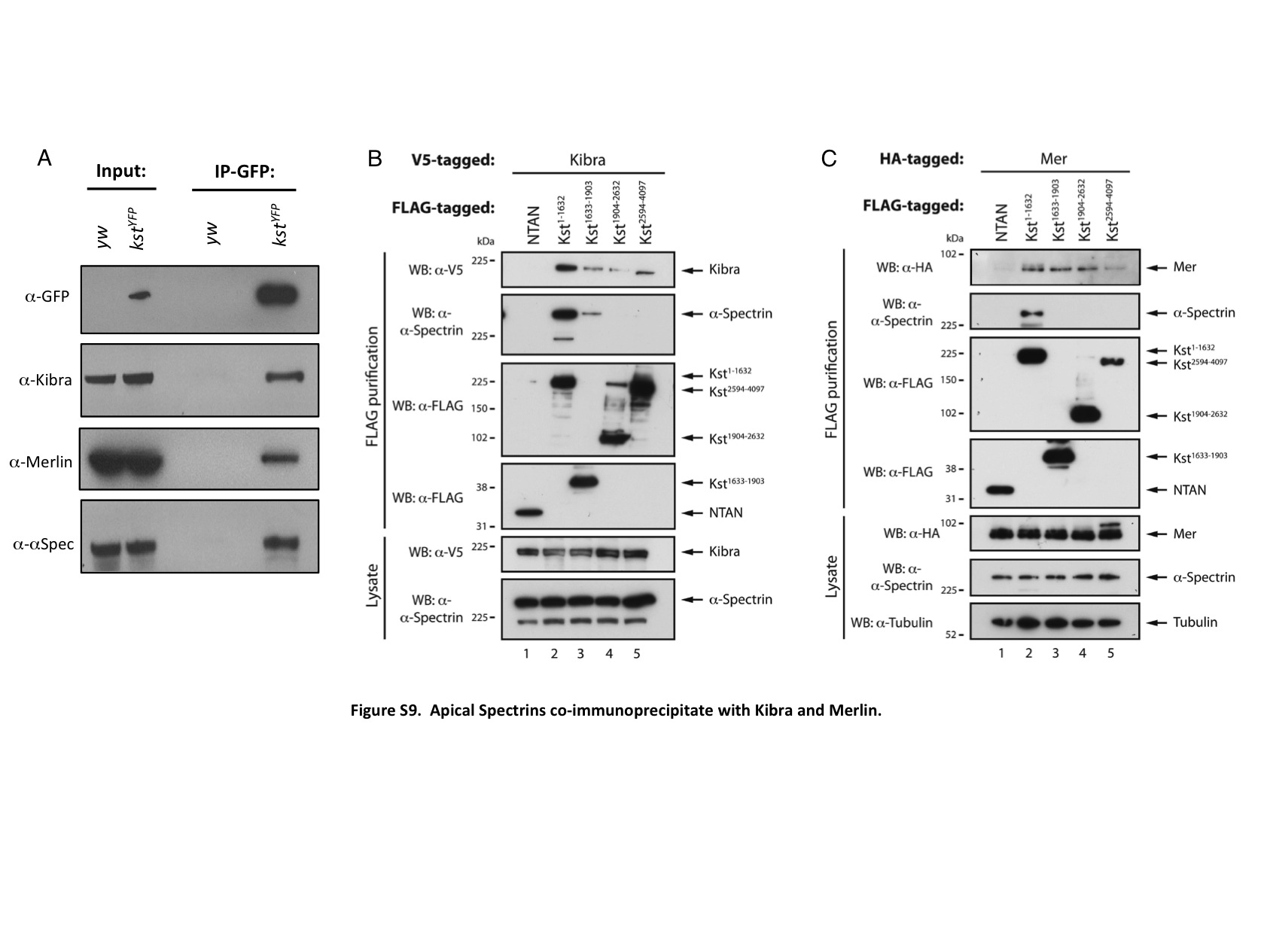

Supplement: Supplementary file 9 [file embj0034-0940-sd9.tif]
