# Supplementary material for: The Spectrin cytoskeleton regulates the Hippo signalling pathway
Source: EMBO J. 2015 Feb 23;34(7):940–54. doi: 10.15252/embj.201489642 (PMC4388601; doi:10.15252/embj.201489642)
Supplement: Supplementary file 10 [file embj0034-0940-sd10.docx]

**Supplementary Materials and methods**

***Drosophila* genotypes used in this study:**

Fig 1A: *w; ey.G4 GMR.G4/+*

Fig 1B: *w; nub.G4/+*

Fig 1C: *w; ey.G4 GMR.G4/UAS.α-specIR* (VDRC 25387)

Fig 1D: *w; nub.G4/UAS.α-specIR*

Fig 1E: *w; ey.G4 GMR.G4/UAS.kstIR* (VDRC 37074)

Fig 1F: *w; nub.G4/UAS.kstIR*

Fig 1G: *w; ey.G4 GMR.G4/UAS.β-specIR* (VDRC 42053)

Fig 1H: *w; nub.G4/UAS.β-specIR*

Fig 1I: *w; ey.G4 GMR.G4/+; UAS.kibIR/+* (Genevet et al, 2010)

Fig 1J: *w; nub.G4/+; UAS.kibIR/+*

Fig 1K: *w; ey.G4 GMR.G4/UAS.α-specIR; UAS.kibIR/+*

Fig 1L: *w; nub.G4/UAS.α-specIR; UAS.kibIR/+*

Fig 1M: *w; ey.G4 GMR.G4/UAS.kstIR; UAS.kibIR/+*

Fig 1N: *w; nub.G4/UAS.kstIR; UAS.kibIR/+*

Fig 1P: *yw ey.flp/w; tub.G4 UAS.GFP/+; FRT82B / FRT82B tub.Gal80*

Fig 1Q: *yw ey.flp/w; tub.G4 UAS.GFP/+; FRT82B kib^32^ / FRT82B tub.Gal80*

Fig 1R: *yw ey.flp/w; tub.G4 UAS.GFP/UAS.α-specIR; FRT82B kib^32^ / FRT82B tub.Gal80*

Fig 2A: *yw*

Fig 2B: *yw ey.flp/w; tub.G4 UAS.GFP/+; FRT82B kib^32^ / FRT82B tub.Gal80*

Fig 2C: *yw ey.flp/w; tub.G4 UAS.GFP/UAS.α-specIR; FRT82B / FRT82B tub.Gal80*

Fig 2D: *yw ey.flp/w; tub.G4 UAS.GFP/UAS.α-specIR; FRT82B kib^32^ / FRT82B tub.Gal80*

Fig 2E: *yw eyflp;; α Spectrin^D445^ FRT80B /ubi.GFPnls FRT80B*

Fig 2F: *yw eyflp;; karst^d11183^ FRT80B / ubi.GFPnls FRT80B*

Fig 2G: *w;* *ex^lacZ^/CyO; hh.G4/+*

Fig 2H: *w; UAS.α-specIR/ex^lacZ^;* *hh.G4/+*

Fig 2I: *w; UAS.kibIR/ex^lacZ^;* *hh.G4/+*

Fig 2J: *w;* *UAS.α-specIR, UAS-kibIR/ex^lacZ^;* *hh.G4/+*

Fig 2K: *w; UAS.hpoIR/ex^lacZ^;* *hh.G4/+* (VDRC 104169)

Fig 3B: *w;; kst-YFP* (DGRC 115-285)

Fig 3C: *w;; kst-YFP*

Fig 3D: *w; nub.G4/+*

Fig 3E: *w; nub.G4 UAS.ex/+*

Fig 3F: *w; nub.G4/UAS.α-specIR*

Fig 3G: *w; nub.G4 UAS.ex/UAS.α-specIR*

Fig 3H: *w; nub.G4/UAS.kstIR*

Fig 3I: *w; nub.G4 UAS.ex /UAS.kstIR*

Fig 4B: *w;* *ex.lacZ*

Fig 4C: *w*

Fig 4D; *w;; kst-YFP*

Fig 5B: *w; nub.G4/+*

Fig 5C: *w; nub.G4 /+ ; UAS.CrbExTM-GFP/+*

Fig 5D: *w; nub.G4 /+ ; UAS.Wts/+*

Fig 5E: *w; nub.G4/UAS.α-specIR*

Fig 5F: *w; nub.G4/UAS.α-specIR ; UAS.CrbExTM-GFP/+*

Fig 5G: *w; nub.G4/UAS.α-specIR ; UAS.Wts /+*

Fig 5H: *w; nub.G4/UAS.kstIR ;*

Fig 5I: *w; nub.G4/UAS.kstIR ; UAS.CrbExTM-GFP/+*

Fig 5J: *w; nub.G4/UAS.kstIR ; UAS.Wts /+*

Fig 5K: *w; nub.G4/UAS.wtsIR*

Fig 5L: *w; nub.G4/UAS.wtsIR ; UAS.CrbExTM-GFP/+*

Fig 5M: *w; nub.G4/UAS.ajubaIR*

Fig 5N: *w; nub.G4/UAS.ajubaIR ; UAS.CrbExTM-GFP/+*

Fig 5P: *w;; wts-GFP* (Rogulja et al, 2014)

Fig 5Q: *w;; wts-GFP*

Fig 6A; *w*

Fig 6B; *ywhsflp; tub.G4 UAS.GFPnls / UAS.yki3SA; FRT82B / FRT82B tub.Gal80*

Fig 6C: *yw hsflp;; karst^d11183^ FRT80B / ubi.GFPnls FRT80B*

Fig 6D: *yw hsflp;; crb^11A22^ FRT82B/ubi.GFPnls FRT82B*

Fig 6E: *yw hsflp;; α Spectrin^e226^ FRT80B /ubi.GFPnls FRT80B*

Fig 6F: *yw β Spectrin^G113^ FRT19A / w ubi.RFPnls FRT19A; hs.flp*

Fig 6G: *w*

Fig 6H: *yw hsflp;; karst^d11183^ FRT80B / ubi.GFPnls FRT80B*

Fig 6I *yw hsflp;; crb^11A22^ FRT82B/ubi.GFPnls FRT82B*

Fig 6J: *yw hsflp;; α-spec^e226^ FRT80B/ubi.GFPnls FRT80B*

Fig 6K: *yw β-spec^G113^ FRT19A /w ubiRFPnls FRT19A; hs.flp*

Fig 6L: w; *ex^lacZ^ / CyO*

Fig 6M: *yw hsflp; ex^lacZ^/+; kst^d11183^ FRT80B / ubi.GFPnls FRT80B*

Fig 6N: *yw hs.flp; ex^lacZ^/+; crb^11A22^ FRT82B/ubi.GFPnls FRT82B*

Fig 6O: *yw hs.flp; ex^lacZ^/+; α-spec^e226^ FRT80B /ubi.GFPnls FRT80B*

Fig 6P: *yw β-spec^G113^ FRT19A / w ubi.RFPnls FRT19A; hs.flp*

Fig 6Q: *w;*

Fig 6R: *yw β-spec^G113^ FRT19A / w ubi.RFPnls FRT19A; hs.flp*

Fig 6S: *yw β-spec^G113^ FRT19A / w ubi.RFPnls FRT19A; hs.flp*

Fig 7A: *w; myo1A.G4; tubGal80^ts^ UAS.GFP /+*

Fig 7B: *w; myo1A.G4 / UAS.kstIR; tubGal80^ts^ UAS.GFP/+*

Fig 7C: *w; myo1A.G4 / UAS.crbIR; tubGal80^ts^ UAS.GFP/+*

Fig 7D: *w; myo1A.G4/ UAS.yki; tubGal80^ts^ UAS.GFP/+*

Fig 7E: *w; myo1A.G4 / UAS.α-specIR; tubGal80^ts^ UAS.GFP/+*

Fig 7F: *w; myo1A.G4/+; UAS.β-specIR/ tubGal80^ts^ UAS.GFP*

Fig 7H: *w; myo1A.G4; DIAP1-HRE-GFP4.3 /+*

Fig 7I: *w; myo1A.G4 / UAS.kstIR; DIAP1-HRE-GFP4.3 /+*

Fig 7J: *w; myo1A.G4 / UAS.α-specIR; DIAP1-HRE-GFP4.3 /+*

Fig 7K: *w; myo1A.G4 / UAS.yki; DIAP1-HRE-GFP4.3 /+*
