# Supplementary material for: The Spectrin cytoskeleton regulates the Hippo signalling pathway
Source: EMBO J. 2015 Feb 23;34(7):940–54. doi: 10.15252/embj.201489642 (PMC4388601; doi:10.15252/embj.201489642)
Supplement: Supplementary file 11 [file embj0034-0940-sd11.docx]

**Figure S1: Redundancy between Crb and Bazooka/Par3 in epithelial cell polarity enables Crb to have a second function as a regulator of wing growth**

(A-F) All UAS.RNAi lines were driven with Hedgehog-Gal4 in the posterior (P) compartment. Scale bars, 250μm (A-C), 50μm (E).

1. Control adult *Drosophila* wing
2. RNAi knockdown of *baz* results in a normal P-compartment with margin bristle defects.
3. RNAi knockdown of *crb* results in overgrowth of the P-compartment.
4. RNAi knockdown of *baz* and *crb* results in lethality.
5. Wing disc showing normal epithelial polarity upon RNAi knockdown of *crb.*
6. Wing disc showing defective epithelial polarity upon RNAi knockdown of both *baz* and *crb* in the P-compartment.

**Figure S2: Knockdown of Crb, PATJ, Kibra, α-Spec, or Kst/β_H_-Spec in the posterior compartment causes a similar compartment overgrowth phenotype**

(A-F) All UAS.RNAi lines were driven with Hedgehog-Gal4 in the posterior (P) compartment. Scale bars, 250μm.

1. Control adult *Drosophila* wing
2. RNAi knockdown of *crb* results in overgrowth of the P-compartment.
3. RNAi knockdown of *PATJ* results in overgrowth of the P-compartment.
4. RNAi knockdown of *kibra* results in overgrowth of the P-compartment.
5. RNAi knockdown of *α-spectrin* results in P-compartment overgrowth.
6. RNAi knockdown of *β_H_-spectrin/karst* results in P-compartment overgrowth.

**Figure S3: Loss of Spectrins induced by RNAi or mutant clones in the wing imaginal disc**

1. Clones expressing *actin.Gal4 UAS.GFP UAS.α-spec-IR* induce RNAi knockdown of α-Spec.
2. Expression of *nub.Gal4* *UAS.α-spec-IR* induces RNAi knockdown of α-Spec in the entire wing pouch.
3. Expression of *nub.Gal4* *UAS.β-spec-IR* induces RNAi knockdown of β-Spec in the entire wing pouch.
4. Clones expressing *actin.Gal4 UAS.GFP UAS.β__-spec/kst-IR* induce RNAi knockdown of β__-Spec/Kst.
5. Clones expressing *actin.Gal4 UAS.GFP UAS.β__-spec/kst-IR* induce RNAi knockdown of β__-Spec/Kst.
6. Clones expressing *actin.Gal4 UAS.GFP UAS.β__-spec/kst-IR* induce RNAi knockdown of β__-Spec/Kst.

Scale bars, 100μm.

**Figure S4: Loss of Spectrins induced by RNAi or mutant clones in the follicular epithelium**

1. Control egg chambers showing normal expression of α-Spec in the entire follicular epithelium through stages 4, 6 and 8.
2. Expression of *GR1.Gal4* *UAS.α-spec-IR* induces RNAi knockdown of α-Spec in the entire follicular epithelium after stage 6.
3. Control egg chambers showing normal expression of β-Spec in the entire follicular epithelium through stages 4, 6 and 8.
4. Expression of *GR1.Gal4* *UAS.β-spec-IR* induces RNAi knockdown of β-Spec in the entire follicular epithelium after stage 6.
5. Clones of *β__-spec/kst*^1^ mutant cells (GFP negative) lose expression of apical β__-Spec/Kst staining.
6. Clones of *β__-spec/kst*^1^ mutant cells (GFP negative) lose expression of apical α-Spec staining.
7. Clones of *β__-spec/kst*^1^ mutant cells (GFP negative) do not affect expression of basolateral β-Spec staining.
8. Clones of *β-spec* mutant cells (GFP negative) lose expression of basolateral but not apical α-Spec.
9. Clones of *β-spec* mutant cells (GFP negative) do not affect expression of apical β__-Spec/Kst staining.
10. Clones of *α-spec* mutant cells (α-Spec negative) lose expression of apical β__-Spec/Kst staining.

Scale bars, 100μm (A-D), 10μm (E-J).

**Figure S5: Spectrins are not required to localise Crb or Ex**

1. Crb localises normally in *α-spectrin* mutant clones marked by the absence of GFP.
2. Ex localises normally in *α-spectrin* mutant clones marked by the absence of GFP.
3. Ex localises normally in *α-spectrin* mutant clones marked by the absence of GFP (Z-section).

Scale bars, 20μm.

**Figure S6: Like Ex, apical α-β_H_ Spectrins act in parallel with the Kibra-Exocyst complex to polarise Crb in follicle cells**

1. Control egg chamber expressing YFP-Karst, which localises to the apical domain of follicle cells. Expanded also localises to the apical domain, similar to YFP-Karst.
2. Kibra and Sec15 co-localise at the apical membrane of follicle cells.
3. Kibra and Sec15 co-localise in intracellular punctae in follicle cells (sub-apical section).
4. Mutation of *β_H_-spectrin/karst^1^* does not affect Crb localisation in follicle cells (GFP negative clone).
5. Mutation of *α-spectrin ^d445^* does not affect Crb localisation in follicle cells (GFP negative clone).
6. RNAi knockdown of *α-spectrin* in all follicle cells with *GR1.Gal4* does not affect Crb localisation.
7. Crb is found at the apical membrane in *sec15^1^* mutant cells at slightly lower levels than wild-type.
8. RNAi knockdown of *α-spectrin* in *sec15^1^* mutant cells results in Crb accumulating in vesicles.
9. RNAi knockdown of *α-spectrin* in *sec15^1^* mutant cells results in Crb accumulating in vesicles (sub-apical section).
10. RNAi knockdown of *β_H_-spectrin/karst* in *sec15^1^* mutant cells results in Crb accumulating in vesicles.
11. RNAi knockdown of *β_H_-spectrin/karst* in *sec15^1^* mutant cells results in Crb accumulating in vesicles (sub-apical section).
12. RNAi knockdown of *α-spectrin* in *kibra^32^* mutant cells results in Crb accumulating in vesicles.
13. RNAi knockdown of *α-spectrin* in *kibra^32^* mutant cells often results in strong overproliferation of cells (MARCM clone affecting all follicle cells).

Scale bars, 20μm (A, B, G, H, J, L, M), 10μm (C-F, I, K).

**Figure S7: α-β Spectrins act in parallel with Kibra to promote border cell migration**

1. Control MARCM clones induced with an FRT82 chromosome and marked by GFP expression. Stage 10 egg chambers.
2. kibra mutant MARCM clones sometimes cause a mild delay in border cell migration.
3. kibra mutant MARCM clones expressing *β_H_-spectrin/karst* RNAi behave identically to kibra mutant clones.
4. kibra mutant MARCM clones expressing *α-spectrin* RNAi often exhibit strongly delayed migration.
5. Quantification of border cell migration delays at stage 10 of oogenesis. N=10-30.
6. Localisation of Spectrins in stage 11 egg chambers.
7. Localisation of Spectrins in border cell clusters, close-up of region boxed in F. β_H_-Spectrin/Karst is expressed in nurse cell membranes but appears to be absent from the border cell cluster.
8. Comparison of Arm and β_H_-Spectrin/Karst expression levels by antibody staining, note similar levels in follicle cells but absence of β_H_-Spectrin/Karst in border cells at stage 10A (prior to centripetal cell migration).
9. Arm and β_H_-Spectrin/Karst antibody staining in border cells, close-up of boxed region in H.

Scale bars, 100μm (A-D), 50μm (F, H), 5μm (G, I).

**Figure S8: Localisation of Spectrins in *Drosophila* and human epithelia.**

1. Localisation of Spectrins in the wing imaginal disc epithelium.
2. Localisation of Spectrins in the follicular epithelium.
3. Localisation of Spectrins in the adult intestinal epithelium.
4. Localisation of Spectrins in human Caco-2 epithelial cells in culture.

Scale bars, 20μm.

**Figure S9: Apical Spectrins co-immunoprecipitate with Kibra and Merlin**

1. In vivo immunoprecipitation with anti-GFP antibodies from either wild-type or Karst-YFP *Drosophila* embryos. Kst-YFP, α-Spectrin, Kibra, and Merlin all co-immunoprecipitate.
2. V5-tagged Kibra was expressed in S2 cells together with various Kst truncations. We find that Kibra co-IPs with the N-terminal region of Kst, which also pulls down endogenous α-Spectrin. Some Kibra was also pulled down with other truncations.
3. V5-tagged Merlin was expressed in S2 cells together with various Kst truncations. We find that Merlin co-IPs with the N-terminal region of Kst, which also pulls down endogenous α-Spectrin. Some Merlin was also pulled down with other truncations.
