# Supplementary material for: The Spectrin cytoskeleton regulates the Hippo signalling pathway
Source: EMBO J. 2015 Feb 23;34(7):940–54. doi: 10.15252/embj.201489642 (PMC4388601; doi:10.15252/embj.201489642)
Supplement: Supplementary file 12 [file embj0034-0940-sd12.pdf]

THE EMBO JOURNAL

Manuscript EMBO-2014-89642

The Spectrin cytoskeleton regulates the Hippo signalling pathway

Georgina C. Fletcher, Ahmed Elbediwy, Ichha Khanal, Paulo S. Ribeiro, Nic Tapon and Barry J. Thompson

Corresponding author: Barry J. Thompson, Cancer Research UK

Review timeline:

Submission date:	29 July 2014
Editorial Decision:	27 August 2014
Revision received:	20 November 2014
Editorial Decision:	07 January 2015
Revision received:	15 January 2015
Accepted:	21 January 2015

Transaction Report:

(Note: With the exception of the correction of typographical or spelling errors that could be a source of ambiguity, letters and reports are not edited. The original formatting of letters and referee reports may not be reflected in this compilation.)

Editor: David del Álamo

1st Editorial Decision 27 August 2014

Thank you for the submission of your manuscript and for your patience while it has been evaluated. We have now received the full set of reports from the referees, which I copy below.

As you can see from their comments, all three referees emphasize the interest and the novelty of linking spectrins to the hippo pathway. They all agree in the necessity for better controls (RNAi experiments, for example) and a more careful interpretation of your genetic analysis, as detailed below. Referee #2 also points out to the fact that your conclusions, particularly the link between membrane tension and the hippo pathway, must be better substantiated before your manuscript can be published in The EMBO Journal.

Given the positive opinions of the referees, I would like to invite you to submit a revised version of the manuscript.

Thank you very much again for the opportunity to consider your work for publication. I look forward to your revision.

© European Molecular Biology Organization

REFEREE COMMENTS

Referee #1:

This is an interesting MS. I like the results and found no major problems in their experiments. I have however major concerns on their interpretation. Fortunately, these can be easily fixed by text changes and better discussion.

They identify a-spectrin as mild inhibitor of Yki function, quantitatively similar to crumbs alleles. Ex.lacZ level raise. They also found genetic interaction with Kibra. However, the interpretation (page 5) that their results show that Spectrins regulate Hippo signaling is not formally correct: Ex.lacZ or DIAP1-GFP read Yki activity (irrespectively of the mechanisms of its activation) and overlapping phenotypes do not necessarily make a pathway.

Figure 3D-I: the interpretation that the Hippo pathway is involved is based on Wts overexpression (but has limited mechanistic value) and Ajuba knockdown. It has been recently shown (Rauskolb et al., 2014) that jub recruits Wts to junctions in a tension-dependent manner. How do we know that warts is actually controlling the cytoskeleton and only indirectly yki activity (downstream of a yet undefined cytoskeletal pathway)? These same authors also cite their own paper Lucas et al., whereby the claim was that hpo/wts directly control actin dynamic, Ena and, as such, border cell migration. How can this pathway not be involved here? Mechanistically, in Rauskolb et al., jub links to a-catenin, that in mammals operates independently of Wts to regulate Yap (Schlegelmilch).
In sum, I expect these authors to have a balanced interpretation and avoid shortcuts in reading their genetics.

It appears that Figure 4 is not central to the present paper. It can be part of a different story on the cell biology of spectrins, sorting and polarity factors, but should be deleted from this MS.

Figure 5: loss of Spectrin, a prototypic actin binding protein, causes changes in cell shape in posterior follicle cells. Isn't this totally expected? How does this connect to membrane tension? There is no experiment supporting this claim.

Please revise your text,: change "....Hippo signaling" with "....Yki activity" in your conclusion statements at the end of each paragraph. And then dedicate efforts in the discussion in proposing different interpretations: a hippo-wts-yki regulation, vs a hippo cytoskeletal control or a mechanical-cytoskeletal-yki pathway.

Figure 6 is very nice. Loss of a-spec or b-spec enhances SC proliferation. Again the interpretation of a feedforward loop on hippo is misplaced. There is not a single evidence - neither in flies nor in mammals - that the hippo pathway is patterned in the intestine (and in many other organs). Hippo may be a fundamental regulatory layer that "sets the tone" of Yki activity, without providing precise instructions to one cell (and not to its neighbor). I was recently reading an interesting discussion from S. Piccolo intestine paper in Cell, where he mentioned some references that crypt shape may pattern yki activity in mammals.Could this be applicable in this case as well? It appears that spectrin may represent a mean to blunt and pattern yki activity, but how could this be solely connected to an unpatterned wts function? There may be entire new worlds to conquer out there and there is no point in such monochord interpretation

The same error appears in Figure 7. As far as I know the mechanical regulation of yki by cell density has been shown to be formally hippo and lats independent by the both the Dupont and follow up papers. Finding Lats phosphorylation down the road of a cytoplasmically relocalized yap does not make lats a primary functional effector, but a reinforcing mechanism for Yki/yap inhibition. Please consider this for your revision.

The present discussion is not particularly engaging. Please make space in discussion by deleting (or shrinking extensively) the discussion on spectrin and mechanosensory functions (second last paragraph, for example). There is little of this in this paper, that fits more with the cytoskeletal control, direct or indirect, of Yki.

Referee #2:

Fletcher and colleagues find that loss of spectrin leads to enhanced growth in several Drosophila tissues (wing, eye, egg follicle, intestine). The evidence presented suggests that mis-regulation of the Hippo pathway is responsible for the growth defects in spectrin compromised tissues. In the wing and eye 'apical spectrin' composed of bHeavy(bH)-Spectrin and a-Spectrin appear to be critical whereas in the ovary and the intestine 'basolateral spectrin' composed of b-Spectrin and a-Spectrin is required for growth control. bH-Spec is a known binding partner of The transmembrane protein Crumbs (Crb), an upstream regulator of Hippo and a binding partner of Expanded (Ex), which acts together with Crb to regulate Hippo activity. Here the authors show that Ex can bind (directly or indirectly) to the N-terminal region of bH-Spec. This together with genetic evidence suggests that Crb and Ex cooperate with apical spectrin to regulate Hippo. Finally, knockdown of Spectrin isoforms in mammalian Caco2 cells also leads to nuclear translocation of YAP in high confluence cells were YAP is normally cytoplasmic, suggesting a loss of Hippo pathway activity.

Collectively, the authors make a compelling case that the spectrin cytoskeleton has an impact on regulating Hippo signaling. The paper represents a survey in going through 5 different tissues but has very little depth. The authors develop a model posing that the ability of the spectrin cytoskeleton to act as a tensor-sensor could explain the impact on Hippo; here high tension would lower the density of Crb-Ex-Hippo/Warts kinases, thus decreasing Hippo signaling activity and promoting growth through nuclear translocation of Yorkie/YAP/TAZ. This model, while interesting is not based in any substantive evidence (what is presented is highly preliminary and circumstantial). The level of analysis would put this paper into a journal like Genetics or similar. More direct support for the tension hypothesis would be needed to make this paper a strong candidate for a top journal.

Moreover:
1) The authors presume that RNAi lines work although when they have no effect it could be that they don't. There may also be issue-specific differences in the effectiveness of RNAi lines. A better characterization of some of the lines needs to be presented. For example, does bH-specRNAi KD bH-Spec::YFP in follicle cells, etc.

2) The logic of genetic interactions needs to be better explained. For example, the enhanced phenotype of kib a-specRNAi (Figure 2) is taken as evidence that spectrin and kib act in parallel whereas the enhanced phenotypes of double KD in Figure 3 are taken as evidence that Ex and spectrin are cooperating in one pathway. A reader not familiar with the details of genetic analysis will be confused.

3) The interaction between kib and a-spec in border cell migration (page 7 and Figure S3) seem to be additive and can therefore not taken as evidence that spectrin regulates Hippo signaling in these cells.

4) Intestine: is the driver myo1A.G4 expressed both in stem cells and enterocytes or only in enterocytes. The authors conclude that 'stem cells' over proliferate but no evidence is presented except that more cell division is observed. Is Crb expressed in the intestine? If not, what's the point of a crb KD?

5) Figure 5: Tanentzapf et al. JCB 2000 reported posterior multilayering in crb mutant follicles. A quantification of these defects would be useful.

6) Figure 4: that Crb localizes to endosomes in spec sec15 or spec kib mutants/KD is not supported by any analysis. Is Crb over-abundant in spec kib double mutants?

7) Figure S5: there seems to be some bH-Spec in border cells.

© European Molecular Biology Organization

Referee #3:

- general summary and opinion about the principle significance of the study, its questions and findings
- specific major concerns essential to be addressed to support the conclusions
- minor concerns that should be addressed
- any additional non-essential suggestions for improving the study (which will be at the author's/editor's discretion)

Summary

In the manuscript titled " The spectrin------- Hippo pathway", Fletcher et al., report the identification of Spectrins as important regulators of the Hippo pathway. They show that Spectrins regulate the cytoskeleton in response of mechanical force, and propose their role as mechanosensors of the Hippo pathway. Fletcher et al., identified both alpha and beta H(heavy) Spectrins in genetic screens for novel growth regulatory genes. The loss-of-function phenotype of alpha and betaH-Spectrin is reminiscent of loss of Crumbs (Crb,another upstream regulator of Hippo). Subsequent analysis in wing, eye, follicular epithelium and border cells, revealed that the alpha- and betaH- Spectrins localize to the apical domain, and genetically interact with kibra. Loss of apical Spectrins caused mild induction of expanded-lacZ (ex-lacZ)- a transcriptional reporter of Yki activity in the Hippo pathway. Biochemical analyses revealed that betaH-Spectrins can exist in a complex with EX, and alpha Spectrin. Spectrins (alpha/betaH) co-localize with EX and Crb to the apical domain, however, the alpha/betaH-Spectrin complex is dispensible for apical localization of EX or Crbs. Genetic interaction analysis places the spectrins parallel or upstream of Ex, as loss of spectrin phenotype is suppressed by overexpression of Ex, or loss of downstream Hippo components like Ajuba. In other tissues - like the follicular epithelium, apical spectrins are involved in Ex-mediated Crb polarization, but not in regulation of membrane tension or Hippo signaling, which is controlled by the basolateral Spectrins. The basolateral spectrins also control Hippo signaling in the intestinal enterocytes. Finally using mammalian cells, Fletcher et al., show that suppression of the mammalian counterparts of alpha /betaH Spectrins influences the nuclear localization of YAP in confluent cells, suggesting that Spectrins are important for sensing cell density by the Hippo pathway.

Major comments:

1. The authors present a loss of function phenotype of alpha/beta-H spectrin using RNAi based transgenes. Although the data presented are interesting, the authors should show the effects of loss of alpha- and beta-H Spectrins using mutant alleles (in mosaics or trans-allelic combinations) e.g., kst1 FRT80B (Fig. 4d) and α-specd445 FRT80B (Fig.4E). Can the loss of function clones produce a similar phenotype as the RNAi based transgenes, for example, in the pupal retina?
The authors should show that loss of alpha- or betaH-spectrin using the RNAi based transgenes has an effect on lowering/abolishing the expression of these genes- e.g., using antibodies or western blotting, or cite previous studies that have confirmed this.

2. Fletcher et al., have shown effects of loss of either alpha or betaH-spectrin on eye and wing growth. However, for the most part, alpha- and betaH-spectrin exist and function as heterodimers in the cells. Therefore, it would be interesting to know what are the effects of simultaneous loss of both alpha and beta-HSpectrin on growth of the wing or eye? Is there a stronger effect on the genetic interaction with Hippo pathway (effect on kibIR and ex-lacZ expression).

3. The authors show that although Ex, Mer or Kib can bind with the apical spectrins, Spectrins are not required for the apical localization of Ex, and loss of the apical spectrins does not modify the over-expression phenotype of Ex, suggesting that the apical spectrins act upstream of Ex. The authors do not mention if additional experiments were performed to test if EX binds the apical Spectrins in-vivo in S2 cells or in other tissues (not just when over-expressing tagged version of these proteins in S2 cells). It is possible that the binding of the N-terminal part of Kst to Ex may not occur in-vivo, or occur only under certain conditions- the authors should comment on these possibilities in the results, and in the discussion.

4. The authors show elegantly that in different tissues the apical and basolateral spectrins affect the Hippo pathway and its functions e.g., with respect to polarization of Crb at the membrane. These are interesting and important findings. However, in epithelial cells like those of wing imaginal discs,

since the apical Spectrins do not function to localize Crb, do they function as mechanical force sensors like in follicular epithelial cells? Does Kst-YFP expression (and cell shape) remain unaltered in crb-/- or kibIR mutant clones?

Minor comments:
In the methods section in the sub-section titled "Immunostaining of ovaries, imaginal discs, and pupal retinas" - rabbit anti-expanded (1:200, gift from A. Laughon, University of Wisconsin-Madison, Madison, WI) is mentioned twice.

1st Revision - authors' response 20 November 2014

We thank all three reviewers for their constructive comments, which we address below:

Referee #1:

This is an interesting MS. I like the results and found no major problems in their experiments. I have however major concerns on their interpretation. Fortunately, these can be easily fixed by text changes and better discussion.

We thank the reviewer for this suggestion. There is currently a strong dogma in the Drosophila field that all regulation of Yki is mediated via the canonical Hpo-Wts cascade. We fully agree that results in mammalian cells clearly show some LATS-independent regulation of YAP/TAZ via the actin cytoskeleton. These two fields have not yet properly reconciled, and our first version was written from the Drosophila-centric perspective. We have now revised our introduction, interpretations and discussion to include the idea of direct regulation of YAP/TAZ via the actin cytoskeleton.

They identify a-spectrin as mild inhibitor of Yki function, quantitatively similar to crumbs alleles. Ex.lacZ level raise. They also found genetic interaction with Kibra. However, the interpretation (page 5) that their results show that Spectrins regulate Hippo signaling is not formally correct: Ex.lacZ or DIAP1-GFP read Yki activity (irrespectively of the mechanisms of its activation) and overlapping phenotypes do not necessarily make a pathway.

To address this point, we have removed the phrase 'Hippo signalling' throughout the results section and replaced it with the phrase 'Yki activity' to make our interpretations more precise. In addition, this allows for the possibility of Hpo-Wts independent regulation of Yki in Drosophila, similar to that observed in mammals.

Figure 3D-I: the interpretation that the Hippo pathway is involved is based on Wts overexpression (but has limited mechanistic value) and Ajuba knockdown. It has been recently shown (Rauskolb et al., 2014) that jub recruits Wts to junctions in a tension-dependent manner. How do we know that warts is actually controlling the cytoskeleton and only indirectly yki activity (downstream of a yet undefined cytoskeletal pathway)? These same authors also cite their own paper Lucas et al., whereby the claim was that hpo/wts directly control actin dynamic, Ena and, as such, border cell migration. How can this pathway not be involved here? Mechanistically, in Rauskolb et al., jub links to a-catenin, that in mammals operates independently of Wts to regulate Yap (Schlegelmilch).
In sum, I expect these authors to have a balanced interpretation and avoid shortcuts in reading their genetics.

To address this point, we add the following sentence to the introduction section:

"Interestingly, regulation of YAP by mechanical stretching and the F-actin cytoskeleton appears to be partly independent of LATS phosphorylation of YAP and must involve an unknown mechanism (Aragona et al, 2013; Dupont et al, 2011)."

We also add the following paragraph to the discussion section:

"Since Spectrins can bind to F-actin, it is possible that force upon Spectrins may also influence the actin cytoskeleton and therefore potentially influence Yki activation independently of Hpo and Wts, as has been shown for the mammalian homologues YAP/TAZ (Aragona et al, 2013; Dupont et al, 2011). Furthermore, Wts has also been shown to influence F-actin polymerisation via regulation of Ena/VASP proteins (Lucas et al, 2013), raising the possibility that canonical Hpo-Wts signalling may also act via regulation F-actin dynamics to control Yki, in addition to direct phosphorylation of the Yki protein by Wts. Further work is necessary to test whether F-actin regulation of Yki occurs in *Drosophila* as it does in mammalian cells."

It appears that Figure 4 is not central to the present paper. It can be part of a different story on the cell biology of spectrins, sorting and polarity factors, but should be deleted from this MS.

We have now removed Figure 4 from the main manuscript and moved it to the supplementary figures.

Figure 5: loss of Spectrin, a prototypic actin binding protein, causes changes in cell shape in posterior follicle cells. Isn't this totally expected? How does this connect to membrane tension? There is no experiment supporting this claim.

We agree that an alteration in cell shapes, consistent with abnormal tension at cell membranes, is an expected phenotype for Spectrin loss based on its known role in red blood cells and C. elegans. However, the cell shape/membrane tension phenotype in Drosophila has not been shown before so we would like to include it here.

Please revise your text,: change "....Hippo signaling" with "....Yki activity" in your conclusion statements at the end of each paragraph. And then dedicate efforts in the discussion in proposing different interpretations: a hippo-wts-yki regulation, vs a hippo cytoskeletal control or a mechanical-cytoskeletal-yki pathway.

As mentioned above, we have removed the phrase 'Hippo signalling' throughout the results section and replaced it with the phrase 'Yki activity' to make our interpretations more precise. In addition, this allows for the possibility of Hpo-Wts independent regulation of Yki in Drosophila, similar to that observed in mammals. The discussion section has also be amended as described above.

Figure 6 is very nice. Loss of a-spec or b-spec enhances SC proliferation. Again the interpretation of a feedforward loop on hippo is misplaced. There is not a single evidence - neither in flies nor in mammals - that the hippo pathway is patterned in the intestine (and in many other organs). Hippo may be a fundamental regulatory layer that "sets the tone" of Yki activity, without providing precise instructions to one cell (and not to its neighbor). I was recently reading an interesting discussion from S. Piccolo intestine paper in Cell, where he mentioned some references that crypt shape may pattern yki activity in mammals.Could this be applicable in this case as well? It appears that spectrin may represent a mean to blunt and pattern yki activity, but how could this be solely connected to an unpatterned wts function? There may be entire new worlds to conquer out there and there is no point in such monochord interpretation

We agree that there is no evidence for Hpo-Wts signalling being patterend in the normal, resting, intestine of the fly (or mammals). What makes the stem cells have higher Yki activity is still not understood in Drosophila or mammals. However, damage to the enterocytes or loss of Wts does lead to increased Yki activity and proliferation in stem cells. We show that loss of Spectrins has the same phenotype as loss of Wts, which simply supports our finding that Spectrins are an important regulator of Yki activity. The fly intestine is a little different from the mammalian intestine (and is in fact more like the bronchial epithelium in character). There are no crypts in the fly gut, it is simply a tube.

The same error appears in Figure 7. As far as I know the mechanical regulation of yki by cell density has been shown to be formally hippo and lats independent by the both the Dupont and follow up papers. Finding Lats phosphorylation down the road of a cytoplasmically relocalized yap does not make lats a primary functional effector, but a reinforcing mechanism for Yki/yap inhibition.

Please consider this for your revision.

We agree fully and have revised the description of this experiment. Plus see the added discussion mentioned above.

The present discussion is not particularly engaging. Please make space in discussion by deleting (or shrinking extensively) the discussion on spectrin and mechanosensory functions (second last paragraph, for example). There is little of this in this paper, that fits more with the cytoskeletal control, direct or indirect, of Yki.

The added discussion mentioned above addresses this point.

Referee #2:

Fletcher and colleagues find that loss of spectrin leads to enhanced growth in several Drosophila tissues (wing, eye, egg follicle, intestine). The evidence presented suggests that mis-regulation of the Hippo pathway is responsible for the growth defects in spectrin compromised tissues. In the wing and eye 'apical spectrin' composed of bHeavy(bH)-Spectrin and a-Spectrin appear to be critical whereas in the ovary and the intestine 'basolateral spectrin' composed of b-Spectrin and a-Spectrin is required for growth control. bH-Spec is a known binding partner of The transmembrane protein Crumbs (Crb), an upstream regulator of Hippo and a binding partner of Expanded (Ex), which acts together with Crb to regulate Hippo activity. Here the authors show that Ex can bind (directly or indirectly) to the N-terminal region of bH-Spec. This together with genetic evidence suggests that Crb and Ex cooperate with apical spectrin to regulate Hippo. Finally, knockdown of Spectrin isoforms in mammalian Caco2 cells also leads to nuclear translocation of YAP in high confluence cells were YAP is normally cytoplasmic, suggesting a loss of Hippo pathway activity.

Collectively, the authors make a compelling case that the spectrin cytoskeleton has an impact on regulating Hippo signaling. The paper represents a survey in going through 5 different tissues but has very little depth. The authors develop a model posing that the ability of the spectrin cytoskeleton to act as a tensor-sensor could explain the impact on Hippo; here high tension would lower the density of Crb-Ex-Hippo/Warts kinases, thus decreasing Hippo signaling activity and promoting growth through nuclear translocation of Yorkie/YAP/TAZ. This model, while interesting is not based in any substantive evidence (what is presented is highly preliminary and circumstantial). The level of analysis would put this paper into a journal like Genetics or similar. More direct support for the tension hypothesis would be needed to make this paper a strong candidate for a top journal.

We thank the reviewer for this comment. To address this point, we provide additional data in support of our model, focussing in depth on the wing imaginal disc where forces stretch cells in a circumferencial pattern around the wing pouch. The new data are presented in the new Figures 4 & 5. Figure 4 shows the correlation between tissue stretching and the expression of the Yki target gene ex.lacZ. It also shows that stretching dilutes Crb and apical Spectrin intensity at cell-cell junctions in a pattern that inversely correlates with the expression of ex.lacZ. This correlation supports the model we propose that force de-clusters Crb complexes. Figure 5 then tests this model by ectopically inducing clustering of Crb complexes and showing that this is sufficient to cause tissue undergrowth, even in the absence of Spectrins. However, the effect of clustering Crb can be reversed by RNAi of Wts, which demonstrates that Crb clustering acts via Wts to regulate tissue size. These data are consistent with the model we propose and we provide a diagram to explain this experiment as clearly as possible. We further show that an alternative model recently published in Cell is unlikely to explain the force-based regulation of Hippo signalling in the wing disc.

Moreover:
1) The authors presume that RNAi lines work although when they have no effect it could be that they don't. There may also be issue-specific differences in the effectiveness of RNAi lines. A better characterization of some of the lines needs to be presented. For example, does bH-specRNAi KD bH-Spec::YFP in follicle cells, etc.

We provide new data in Figure S3 and S4 to confirm that our RNAi lines do work and knockdown their target proteins, as expected.

2) The logic of genetic interactions needs to be better explained. For example, the enhanced phenotype of kib a-specRNAi (Figure 2) is taken as evidence that spectrin and kib act in parallel whereas the enhanced phenotypes of double KD in Figure 3 are taken as evidence that Ex and spectrin are cooperating in one pathway. A reader not familiar with the details of genetic analysis will be confused.

We add new text to explain this analysis in the results section. The enhancement of phenotypes in kib, ex double mutants or kib a-spec double mutants are similar and suggest that a-spec works with Ex in the same pathway. In Figure 3, overexpression of Ex causes a small wing even in the absence of Spectrins, suggesting Spectrins act upstream of Ex.

3) The interaction between kib and a-spec in border cell migration (page 7 and Figure S3) seem to be additive and can therefore not taken as evidence that spectrin regulates Hippo signaling in these cells.

We agree that this phenotype appears to be an additive one, but all we seek to show is that these two proteins act in parallel to promote border cell migration. The fact that they have a role in border cell migration would be expected of proteins involved in Hippo signalling.

4) Intestine: is the driver myo1A.G4 expressed both in stem cells and enterocytes or only in enterocytes. The authors conclude that 'stem cells' over proliferate but no evidence is presented except that more cell division is observed. Is Crb expressed in the intestine? If not, what's the point of a crb KD?

In the fly intestine, almost all cell divisions are those of stem cells. There is controversy about whether daughter cells then undergo additional divisions to sometimes produce enteroendocrine cells rather than enterocytes, but these possible divisions are only a minority so can be disregarded for the purposes of our analysis. Stem cells are also characterised by the expression of DIAP1-HRE-GFP, which is increased upon spectrin knockdown in the gut. The point of the Crb-RNAi experiment is to show that it has no phenotype in the gut. We find that some cells in the gut do express Crb (although not the entire gut).

5) Figure 5: Tanentzapf et al. JCB 2000 reported posterior multilayering in crb mutant follicles. A quantification of these defects would be useful.

We have quantified the crb mutant phenotype, which exhibits multilayering in around 10% of cases. In contrast the a-Spec mutant has a 95% penetrant multilaying phenotype. These quantifications have been added to the text of the Results section.

6) Figure 4: that Crb localizes to endosomes in spec sec15 or spec kib mutants/KD is not supported by any analysis. Is Crb over-abundant in spec kib double mutants?

This data has now been moved to the supplementary materials section. Since Crb is a transmembrane protein, we assume that any intracellular punctae of Crb staining must be either endosomal or vesicular in character. We have revised the text to clarify this point. We have not measured the degree of accumulation of Crb in spec kib double mutants so we cannot make a statement about abundance of Crb.

7) Figure S5: there seems to be some bH-Spec in border cells.

This bH-Spec signal is from the nurse cell plasma membranes that are closely associated with the border cell cluster.

Referee #3:

Summary

In the manuscript titled " The spectrin------- Hippo pathway", Fletcher et al., report the identification of Spectrins as important regulators of the Hippo pathway. They show that Spectrins regulate the cytoskeleton in response of mechanical force, and propose their role as

mechanosensors of the Hippo pathway. Fletcher et al., identified both alpha and beta H(heavy) Spectrins in genetic screens for novel growth regulatory genes. The loss-of-function phenotype of alpha and betaH-Spectrin is reminiscent of loss of Crumbs (Crb,another upstream regulator of Hippo). Subsequent analysis in wing, eye, follicular epithelium and border cells, revealed that the alpha- and betaH- Spectrins localize to the apical domain, and genetically interact with kibra. Loss of apical Spectrins caused mild induction of expanded-lacZ (ex-lacZ)- a transcriptional reporter of Yki activity in the Hippo pathway. Biochemical analyses revealed that betaH-Spectrins can exist in a complex with EX, and alpha
Spectrin. Spectrins (alpha/betaH) co-localize with EX and Crb to the apical domain, however, the alpha/betaH-Spectrin complex is dispensible for apical localization of EX or Crbs. Genetic interaction analysis places the spectrins parallel or upstream of Ex, as loss of spectrin phenotype is suppressed by overexpression of Ex, or loss of downstream Hippo components like Ajuba. In other tissues - like the follicular epithelium, apical spectrins are involved in Ex-mediated Crb polarization, but not in regulation of membrane tension or Hippo signaling, which is controlled by the basolateral Spectrins. The basolateral spectrins also control Hippo signaling in the intestinal enterocytes. Finally using mammalian cells, Fletcher et al., show that suppression of the mammalian counterparts of alpha /betaH Spectrins influences the nuclear localization of YAP in confluent cells, suggesting that Spectrins are important for sensing cell density by the Hippo pathway.

Major comments:
1. The authors present a loss of function phenotype of alpha/beta-H spectrin using RNAi based transgenes. Although the data presented are interesting, the authors should show the effects of loss of alpha- and beta-H Spectrins using mutant alleles (in mosaics or trans-allelic combinations) e.g., kst1 FRT80B (Fig. 4d) and α-specd445 FRT80B (Fig.4E). Can the loss of function clones produce a similar phenotype as the RNAi based transgenes, for example, in the pupal retina?

The authors should show that loss of alpha- or betaH-spectrin using the RNAi based transgenes has an effect on lowering/abolishing the expression of these genes- e.g., using antibodies or western blotting, or cite previous studies that have confirmed this.

We provide new data in Figs S3 and S4 to show that our RNAi lines do work well in multiple epithelial tissues. We also provide new data to show kst1 and aspec mutant pupal retina phenotypes, which are the same as the RNAi phenotypes.

2. Fletcher et al., have shown effects of loss of either alpha or betaH-spectrin on eye and wing growth. However, for the most part, alpha- and betaH-spectrin exist and function as heterodimers in the cells. Therefore, it would be interesting to know what are the effects of simultaneous loss of both alpha and beta-HSpectrin on growth of the wing or eye? Is there a stronger effect on the genetic interaction with Hippo pathway (effect on kibIR and ex-lacZ expression).

We don't see any enhancement of the a-spec RNAi phenotype when co-expressed with kst RNAi. This is consistent with the heterodimer model. We also provide new data in Fig S4 to show that apical a-Spec depends on kst and basolateral a-Spec depends on beta-spec, as predicted by the heterodimer model.

3. The authors show that although Ex, Mer or Kib can bind with the apical spectrins, Spectrins are not required for the apical localization of Ex, and loss of the apical spectrins does not modify the over-expression phenotype of Ex, suggesting that the apical spectrins act upstream of Ex. The authors do not mention if additional experiments were performed to test if EX binds the apical Spectrins in-vivo in S2 cells or in other tissues (not just when over-expressing tagged version of these proteins in S2 cells). It is possible that the binding of the N-terminal part of Kst to Ex may not occur in-vivo, or occur only under certain conditions- the authors should comment on these possibilities in the results, and in the discussion.

We provide new data to confirm that Mer and Kib bind to apical Spectrins in vivo. Endogenous YFP-tagged Kst (knock-in) was pulled down from embryos and Mer and Kib come with it.

4. The authors show elegantly that in different tissues the apical and basolateral spectrins affect the Hippo pathway and its functions e.g., with respect to polarization of Crb at the membrane. These

are interesting and important findings. However, in epithelial cells like those of wing imaginal discs, since the apical Spectrins do not function to localize Crb, do they function as mechanical force sensors like in follicular epithelial cells? Does Kst-YFP expression (and cell shape) remain unaltered in crb-/- or kibIR mutant clones?

We provide new data (Figs 4 and 5) to support the notion that apical Spectrins function as mechanical force sensors in the wing imaginal disc. Published work shows Crb is required to localise Kst in the eye, but not in tissues where Crb acts redundantly with Baz/Par-3 to define the apical domain, such as imaginal discs or follicle cells.

Minor comments:
In the methods section in the sub-section titled "Immunostaining of ovaries, imaginal discs, and pupal retinas" - rabbit anti-expanded (1:200, gift from A. Laughon, University of Wisconsin-Madison, Madison, WI) is mentioned twice.

Corrected.

2nd Editorial Decision 07 January 2015

Thank you for the submission of your revised manuscript to The EMBO Journal and please accept my apologies for the unexpected delay in responding due to the holiday break. As you will see below, referees consider that you have properly dealt with the main concerns originally raised in the review process, and therefore I am writing with an 'accept in principle' decision, which means that I will be happy to formally accept your manuscript for publication once a few more issues have been addressed.

Your study was sent back to former referees #2 and #3, who as I said now believe that all major concerns have been addressed and your manuscript is almost ready for publication (see below). Only a few minor issues will still require your attention -mostly clarifications and further discussion of certain aspects of your data as pointed out by referee #2- but no further experimental evidence is required. Referee #2 also suggests the inclusion of evidence proving Crb expression in the intestine, which I believe is pertinent given that you already have it and can be easily added to the supplementary information. It will however not be a condition for the acceptance of your manuscript as it does not affect any fundamental conclusion.

Browsing through the manuscript myself I have also noticed a few small issues with data presentation. Micrographs throughout the manuscript (including supplementary data) lack scale bars, which we require for clarity. Furthermore, the statistical analysis of the results presented in panels 1O, 5O and 7G requires a more detailed description. As a guide, statistical analyses must be described either in the Materials and Methods section or in the legend of the figure to which they apply, and will include a definition of the error bars used and the number of independent experiments performed. The statistical significance analysis tool used, if any, must be also clearly stated.

If you have any questions or need any further input, please do not hesitate to contact me.

Thank you very much for your patience and my apologies again for this unacceptable delay. I am looking forward to seeing the final version of your manuscript. Congratulations in advance for a successful publication.

REFEREE COMMENTS

Referee #2:

The authors have significantly improved the paper in response to reviewer comments. The evidence that the spectrin cytoskeleton is involved in Hippo signaling is quite clear. Whether it acts as a

© European Molecular Biology Organization

tension sensor that operates through determining the density of Crb in the membrane remains a somewhat speculative model, but it's certainly an interesting one that will stimulate further research.

Minor points:
- The authors identify Crb distribution in the follicular epithelium as endosomal or vesicular without evidence that Crb is actually endosomal. And of course endosomes are also vesicles. So why not say just 'vesicular'.

- Its 'follicular epithelium' not 'follicle cell epithelium'.

- The authors say in two cases that RNAi of gene X in a tissue has no effect, and then follow this up by saying that the gene is not expressed in that tissue (e.g. crb in the intestine). What's the point of that? In the case of crb the authors claim that the gene is expressed in some cells of the intestine in the rebuttal. This should be shown as it has not been reported previously, and effective KD of that expression should be documented.

- page 6: The correct ref. for CrbExtraTM::GFP is Pellikka et al., 2003.

Referee #3:

The revised manuscript adequately addresses the concerns raised by this referee .

The authors have revised the manuscript to include the additional findings that add to the knowledge in the field .
